# Temporal Differentiation of Nonperipheral Washout Refines Prognostic Stratification in HCC

**DOI:** 10.1111/liv.70838

**Published:** 2026-08-25

**Authors:** Yingyi Wu, Junhan Pan, Ting Yang, Hong Wei, Guzalinu Wufuer, Tianyi Xia, Yuanan Wu, Yanyan Zhang, Hui‐chuan Sun, Shenghong Ju, Feng Chen, Maxime Ronot, Bin Song, Hanyu Jiang, Yanshu Wang

**Affiliations:** ^1^ Department of Radiology West China Hospital, Sichuan University Chengdu China; ^2^ Jiangsu Key Laboratory of Molecular and Functional Imaging, Department of Radiology, Zhongda Hospital, School of Medicine Southeast University Nanjing China; ^3^ Big Data Research Center University of Electronic Science and Technology of China Chengdu China; ^4^ Department of Radiology Beijing Youan Hospital, Capital Medical University Beijing China; ^5^ Department of Hepatobiliary Surgery and Liver Transplantation, Liver Cancer Institute Zhongshan Hospital Fudan University Shanghai China; ^6^ Department of Radiology The First Affiliated Hospital, Zhejiang University School of Medicine Hangzhou China; ^7^ UMR 1149, CRI, Paris & Service de Radiologie, Hôpital Beaujon, APHP.Nord Université Paris Cité Clichy France; ^8^ Department of Radiology Sanya People's Hospital Sanya China

**Keywords:** hepatocellular carcinoma, magnetic resonance imaging, prognosis, washout

## Abstract

**Background and Aims:**

Nonperipheral washout is a major imaging feature of hepatocellular carcinoma (HCC) with prognostic value, but whether its temporal differentiation can refine prognostic stratification remains understudied. This study aimed to investigate the prognostic implications of nonperipheral washout timing in HCC.

**Methods:**

This multicentre, retrospective cohort study included patients who underwent curative resection for single BCLC stage 0/A HCC and preoperative extracellular contrast agent–enhanced MRI at seven tertiary centres (March 2011 to April 2023). Three masked radiologists independently evaluated nonperipheral washout patterns, which were classified as early (present in the portal venous phase), late (present only in the delayed phase), or absent. Early recurrence‐free survival (eRFS; ≤ 2 years) and 5‐year RFS were assessed using Kaplan–Meier and Cox regression analyses.

**Results:**

A total of 611 patients (median age, 55 years; 520 men) were included. Early, late, and no washout were observed in 367 (60.1%), 95 (15.5%), and 149 (24.4%) patients, respectively. Inter‐ and intra‐reader agreements were substantial to excellent (*κ* range, 0.67–0.94). Early washout was associated with microvascular invasion (*p* = 0.01) and Edmondson–Steiner G3/4 (*p* = 0.002). The eRFS rates were 67.3%, 82.1%, and 85.2% for the early, late, and no‐washout groups (*p* < 0.001); the 5‐year RFS rates were 53.4%, 62.1%, and 71.8% (*p* = 0.002). In multivariable Cox analysis adjusting for factors, early washout independently predicted eRFS (HR, 1.86; *p* < 0.001) and 5‐year RFS (HR, 1.46; *p* = 0.006).

**Conclusions:**

In patients undergoing curative resection for single BCLC 0/A HCC, temporal differentiation of nonperipheral washout refines prognostic stratification, with early washout identifying patients at higher risk of recurrence.

## Lay Summary

In patients who underwent curative resection for a single early‐stage hepatocellular carcinoma, this study found that the timing of ‘washout’ on preoperative contrast‐enhanced MRI carries significant prognostic value. Tumours showing early nonperipheral washout, visible during the portal venous phase, were more likely to exhibit aggressive pathological features. Consequently, these patients faced a higher risk of cancer recurrence after surgery. Evaluating early washout on routine MRI may therefore help doctors more accurately identify patients who need closer postoperative monitoring.

## 1 Introduction

Hepatocellular carcinoma (HCC) is unique among solid malignancies in that it can be diagnosed noninvasively based solely on characteristic imaging features in high‐risk populations, without the need for histopathological confirmation [1]. Nonperipheral washout, defined as nonperipherally visualized reduction in enhancement from earlier to later phase resulting in hypoenhancement relative to liver parenchyma in extracellular phases, is a core imaging feature supporting this noninvasive diagnosis [2, 3]. In MRI with extracellular contrast agents (ECAs), nonperipheral washout can be assessed in either the portal venous phase (PVP) or the delayed phase (DP). Still, up to 30% of HCCs exhibit this feature only in the DP [4]. Despite this temporal heterogeneity, all major guidelines treat nonperipheral washout in either the PVP or the DP as equivalent for diagnosing HCC [2, 5, 6, 7].

Beyond its diagnostic utility, emerging evidence suggests that nonperipheral washout is also closely associated with tumour biology and holds substantial prognostic significance. For instance, the presence of nonperipheral washout has been linked to aggressive pathological characteristics and unfavourable clinical outcomes, including poorer differentiation [8, 9], higher rates of microvascular invasion (MVI) [10, 11], the presence of the vessels‐encapsulating tumour clusters pattern [12], and increased recurrence following curative resection [13, 14, 15].

However, similar to the diagnostic context, most previous studies treated nonperipheral washout in either the PVP or the DP as prognostically equivalent. This assumption, that differing onset phases of nonperipheral washout carry similar prognostic implications, may be insufficiently supported, given that these temporal patterns may reflect distinct tumour vasculatures that influence HCC outcomes [7, 16]. Therefore, delineating the clinical, histopathologic, and prognostic differences between nonperipheral washout observed in the PVP versus the DP may help understand the biological meaning underlying this temporal heterogeneity, refine risk stratification, and inform personalized treatment strategies in HCC. Nonetheless, studies specifically addressing this issue remain limited.

Therefore, this study aimed to evaluate the clinical, histopathologic, and prognostic significance of nonperipheral washout observed in the PVP (i.e., early washout) compared with that observed exclusively in the DP (i.e., late washout) among patients who underwent curative liver resection for single Barcelona Clinic Liver Cancer (BCLC) stage 0/A HCC.

## 2 Methods

### 2.1 Patients

This multicentre, retrospective cohort study was conducted in seven tertiary‐care referral hospitals. The study protocol received approval from the institutional review boards of all participating centres, and the requirement for written informed consent was waived.

**FIGURE 1:**
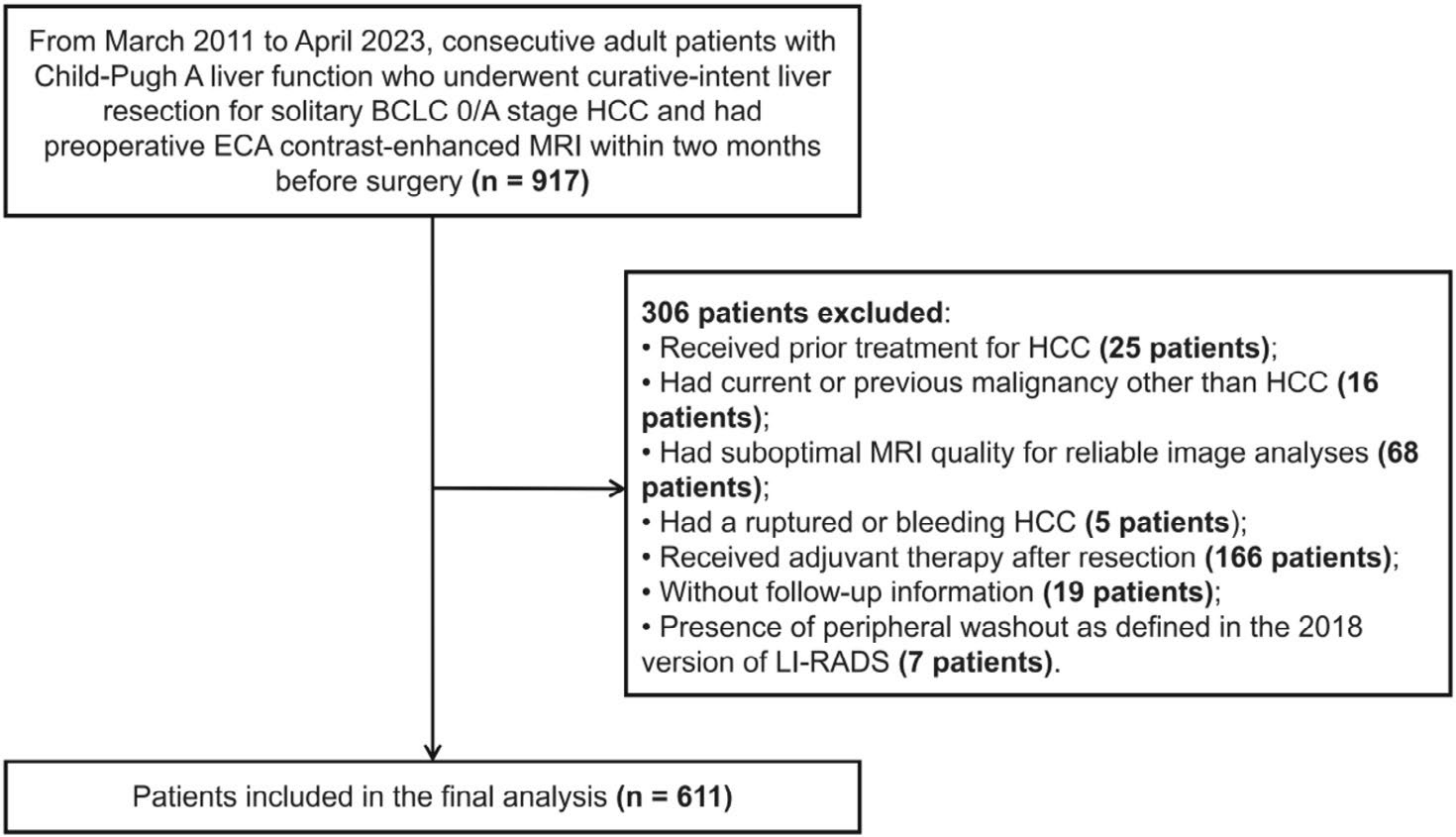
Study flowchart. BCLC = Barcelona Clinic Liver Cancer, ECA = extracellular contrast agent, HCC = hepatocellular carcinoma, LI‐RADS = Liver Imaging Reporting and Data System.

Between March 2011 and April 2023, consecutive patients who underwent curative R0 liver resection for HCC were identified based on the following inclusion criteria: (a) age ≥ 18 years, (b) with Child–Pugh A liver function, (c) had pathologically confirmed HCC, and were clinically staged as BCLC 0/A with an imaging‐assessed single lesion, and (d) underwent ECA‐enhanced MRI within 2 months before resection. The exclusion criteria were: (a) prior treatment for HCC, (b) current or previous malignancy other than HCC, (c) suboptimal MRI quality for reliable image analyses (e.g., severe artefact, non‐standardized acquisition protocol), (d) ruptured or bleeding HCC on imaging or pathology, (e) receipt of adjuvant therapy after resection, (f) without follow‐up information, or (g) presence of peripheral washout as defined in the version 2018 Liver Imaging Reporting and Data System (LI‐RADS) [2]. Notably, 394 patients in this study have been reported previously [17]. While the prior work focused on the clinicohistopathologic correlations and prognostic implications of MRI‐assessed intratumoral fat in HCCs, the current work aimed to investigate the clinicohistopathologic correlations and prognostic implications of the timing of nonperipheral washout on ECA‐MRI. Patient inclusion and exclusion are illustrated in Figure 1.

Resection was performed using open, laparoscopic, or robotic approaches by hepatobiliary surgeons with 5 to over 20 years of experience in liver surgery at each participating centre. Resection type (minimally invasive or open surgery), extent (major or minor), and use of anatomical resection were determined at the surgeons' discretion according to tumour burden, location, and estimated residual liver volume. Intraoperative ultrasonography was routinely employed to evaluate minute intrahepatic tumours.

Baseline clinical information (e.g., age, sex, chronic liver disease aetiologies) and laboratory results within 2 weeks before resection were collected. Data of the tumour (i.e., Edmondson–Steiner grade, MVI, the macrotrabecular‐massive [MTM] subtype) [18, 19], and liver cirrhosis were obtained from routine postoperative histopathologic reports.

### 2.2 MRI Acquisition and Analysis

Preoperative MRI examinations were performed on various 1.5‐T or 3.0‐T systems using standard liver protocols with ECAs (as detailed in Appendix S1 and Table S1). All MRI examinations were performed in compliance with the technical requirements of the LI‐RADS guidelines, including PVP imaging acquired approximately 60 s after contrast administration and DP imaging obtained approximately 3 min after contrast injection.

After one radiologist (Yanshu Wang, 5 years of experience) de‐identified the images and reviewed the histopathology and follow‐up data, the anonymized and randomized MRIs were centrally and independently assessed by three additional fellowship‐trained abdominal radiologists (Hanyu Jiang, Yingyi Wu, and Tinng Yang, with 9, 4, and 5 years of experience in liver imaging, respectively). The reviewers were aware of the HCC diagnosis but blinded to all clinicopathologic and follow‐up information.

**FIGURE 2:**
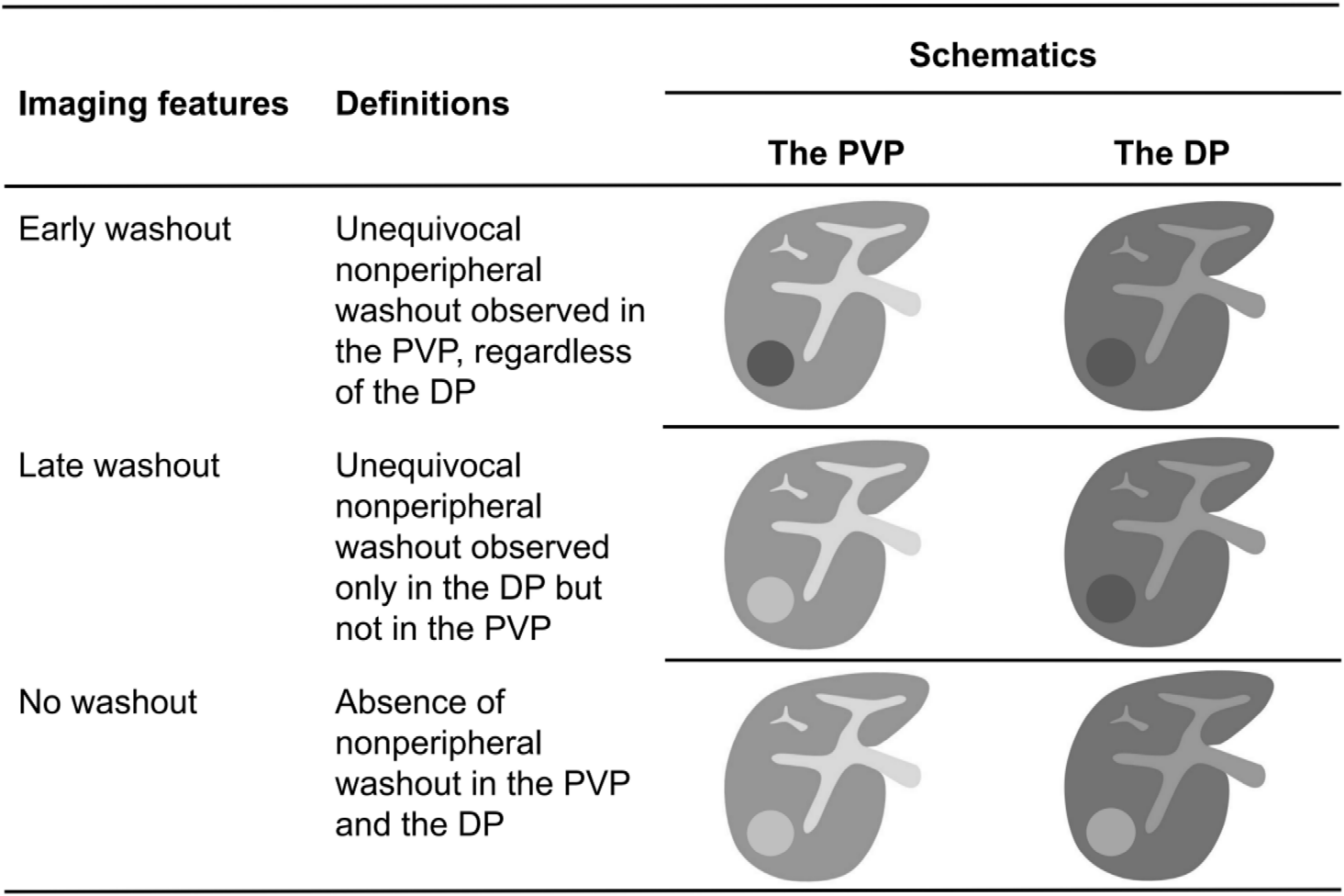
Definitions and schematic illustrations for different nonperipheral patterns. DP = delayed phase, PVP = portal venous phase.

Before the formal image analyses, all readers completed a hands‐on training session using 20 non‐study HCC cases to standardize interpretations. Nonperipheral washout was defined per LI‐RADS version 2018 as a reduction in enhancement (in whole or in part) from earlier to later phase resulting in hypoenhancement relative to liver parenchyma. The phase‐based temporal patterns of nonperipheral washout were classified as follows: (a) early washout: unequivocal washout observed in the PVP, regardless of findings in the DP; (b) late washout: unequivocal washout observed only in the DP but not in the PVP; and (c) no washout: absence of washout in the PVP and DP. To minimize potential recall bias, a three‐round reading protocol with at least 4‐week intervals between rounds was implemented (illustrated in Figures 2 and S1, and detailed in Appendix S2). In the first, second, and third rounds, nonperipheral washout was assessed using randomized and anonymized images from (a) the pre‐contrast phase, AP (arterial phase), and PVP, (b) the pre‐contrast phase, AP and DP, as well as (c) the pre‐contrast phase, AP, PVP, and DP, respectively. After completing the first two rounds, each reviewer synthesized their phase‐specific assessments to determine a temporal pattern of nonperipheral washout (early, late, or no washout), referred to hereafter as the ‘synthesized washout pattern’. Following the third round, reviewers assigned the temporal pattern of nonperipheral washout based solely on the third‐round evaluation, referred to hereafter as the ‘observed washout pattern’.

In addition to the temporal patterns of nonperipheral washout, the reviewers also evaluated 34 imaging features that have well‐established prognostic relevance or may influence the interpretation of nonperipheral washout, including (a) other LI‐RADS version 2018 imaging features [5, 20], (b) previously‐reported prognostic imaging features (e.g., intratumoral artery, PVP peritumoral hypoenhancement) [21, 22, 23], and (c) chronic liver disease‐related imaging features (e.g., cirrhosis, liver steatosis). In accordance with standard LI‐RADS criteria, the LI‐RADS high‐risk population was defined as patients with cirrhosis, chronic hepatitis B virus (HBV) infection, or a prior history of HCC. Detailed definitions of these imaging features are summarized in Table S2. Disagreements among the reviewers were resolved by majority vote or by consulting a senior radiologist with over 20 years of experience in liver MRI. The consensus ‘observed washout pattern’ derived from the third round of evaluation was used for all subsequent clinicopathological comparisons and survival analyses.

### 2.3 Follow‐Up and Endpoints

All patients were regularly followed up postoperatively with serum α‐fetoprotein and contrast‐enhanced imaging (ultrasound, CT, or MRI) at 1 month, then every 3 to 6 months for the first 2 years, and every 6 to 12 months thereafter according to the guidelines [24]. Follow‐up continued until death or the last contact date recorded at each centre (up to November 30, 2024).

The primary endpoint was early recurrence‐free survival (eRFS), defined as the time from resection to the first documented recurrence (intrahepatic HCC, tumour‐in‐vein, or distant metastasis) or death from any cause within 2 years after surgery. Patients without events were censored at the last follow‐up date or 2 years, whichever occurred first. We selected this endpoint because early recurrence has been reported predominantly driven by intrahepatic metastasis from the primary tumour and therefore serves as a surrogate marker of aggressive tumour biology [5]. 5‐year RFS was considered the secondary endpoint since follow‐up beyond 5 years was, in general, more frequently performed in the primary care setting rather than in tertiary referral hospitals.

### 2.4 Statistical Analysis

Statistical analyses are detailed in Appendix S3. In brief, categorical variables were compared using the *χ* ^2^ test or Fisher's exact test, as appropriate; when the overall test was significant, pairwise comparisons were performed with Bonferroni adjustment. Non‐normally distributed continuous/ordinal variables were compared among groups using the Kruskal–Wallis test; when the overall test was significant, pairwise comparisons were performed using Dunn's test with Bonferroni adjustment.

Inter‐rater agreement among the three reviewers was assessed using Fleiss's kappa value for binary imaging features, the intraclass correlation coefficient (ICC) for continuous imaging features, and a weighted kappa value for categorical imaging features. Intra‐rater agreement within each reviewer was assessed using Cohen's kappa value for binary imaging features, ICC for continuous imaging features, and weighted kappa value for categorical imaging features.

Survival outcomes were estimated using the Kaplan–Meier method and compared with the log‐rank test, with a Bonferroni adjustment for multiple comparisons. Uni‐ and multi‐variable Cox regression analyses were performed to investigate the predictive value of nonperipheral washout patterns for eRFS and 5‐year RFS while adjusting for established clinical (e.g., serum alpha‐fetoprotein), radiological (e.g., intratumoral artery), and histopathologic (e.g., MVI) prognostic factors. Sensitivity analyses were conducted in patients with available satellite‐nodule data and in those meeting LI‐RADS high‐risk criteria. Predefined subgroup analyses were performed using multivariable Cox regression for patients with different α‐fetoprotein levels, MRI field strengths, tumour sizes, liver cirrhosis, steatosis, enhancing capsule status, Edmondson–Steiner grade, MVI status, as well as resection margin width, extent, and type. To ensure statistical reliability and prevent model overfitting in smaller subgroups, a parsimonious set of the most robust confounders was used for adjustment. Analyses were omitted for subgroups with fewer than five events to ensure statistical reliability. Fine–Gray competing‐risk models were additionally used for recurrence‐specific analyses, treating death as a competing event.

The impacts of MRI field strength, cirrhosis, liver steatosis, enhancing capsule, and mosaic architecture on the interpretation of nonperipheral washout patterns were evaluated using the chi‐squared test [25, 26].

All analyses were conducted using R (version 4.4.1) or MedCalc (version 20.112), with a two‐tailed *p* < 0.05 considered statistically significant.

This study was designed and reported in accordance with the STROBE (Strengthening the Reporting of Observational Studies in Epidemiology) guidelines. The completed STROBE checklist is provided in the Supplementary Appendix 2.

## 3 Results

### 3.1 Patients

A total of 611 patients (median age, 55 years; IQR, 47–63 years; 520 [85.1%] men) were included (characteristics summarized in Table 1). In brief, chronic HBV infection was the dominant aetiology (536/611, 87.7%), and cirrhosis was observed in 347 (56.8%) patients. The median tumour size was 3.2 cm (IQR, 2.2–4.9 cm), and 491 (80.4%) patients were classified as BCLC A stage.

**TABLE 1:** Baseline clinical, histopathologic, surgical characteristics, and LI‐RADS category distribution of the included patients.

Characteristics	Total (*n* = 611)
Patient demographics	
Age (years)	55.0 (47.0, 63.0)
Sex, male	520 (85.1)
Chronic liver disease^a^	
Hepatitis B virus	536 (87.7)
Hepatitis C virus	11 (1.8)
Alcohol use	27 (4.4)
MASLD	13 (2.1)
Cryptogenic	46 (7.5)
Other	1 (0.2)
LI‐RADS high‐risk status	567 (92.8)
ALBI grade	
1	469 (76.8)
2	142 (23.2)
Tumour burden	
Tumour size (cm)	3.2 (2.2, 4.9)
BCLC stage, 0	120 (19.6)
Laboratory results	
α‐fetoprotein (> 400 ng/mL)	147 (24.1)
AST (U/L)	31.0 (24.0, 43.0)
ALT (U/L)	32.2 (22.4, 49.5)
Total bilirubin (μmol/L)	14.3 (10.6, 19.1)
Albumin (g/L)	43.3 (40.1, 46.1)
Creatinine (μmol/L)	73.0 (65.0, 83.4)
ALP (U/L)	83.0 (68.0, 101.0)
GGT (U/L)	43.0 (24.0, 79.0)
Platelet count (×10^9^/L)	128.0 (86.5, 169.0)
Histopathologic features	
Cirrhosis	347 (56.8)
Edmondson–Steiner grade	
I/II	406 (66.4)
III/IV	205 (33.6)
Microvascular invasion	215 (35.2)
MTM subtype^b^	63 (18.3)
Satellite nodules^c^	26 (6.3)
Surgical information	
Open surgery^d^	423 (69.2)
Major hepatectomy^e^	68 (11.1)
LI‐RADS category^f^	
LR‐4	54 (9.0)
LR‐5	473 (84.1)
LR‐M	40 (6.9)

*Note:* Continuous variables are expressed as medians with IQRs in parentheses, while categorical variables are expressed as numbers with percentages in parentheses.

Abbreviations: ALBI = albumin–bilirubin, ALP = alkaline phosphatase, ALT = alanine aminotransferase, AST = aspartate aminotransferase, BCLC = Barcelona Clinic Liver Cancer, GGT = gamma‐glutamyl transferase, LI‐RADS = Liver Imaging Reporting and Data System, MASLD = metabolic dysfunction‐associated steatotic liver disease, MTM = macrotrabecular massive.

^a^ More than one aetiology could be present for each patient.

^b^ Data are presented for 345 patients with available histopathologic subtyping.

^c^ Data are presented for 411 patients with available histopathologic results.

^d^ All patients in this cohort underwent either laparoscopic or open surgery.

^e^ The extent of resection was categorized as major resection (resection of ≥ 3 segments according to the Couinaud classification) or minor resection (resection of < 3 segments according to the Couinaud classification).

^f^ LI‐RADS categories were reported only for patients with the LI‐RADS high‐risk status (*n* = 567).

### 3.2 Inter‐ and Intra‐Rater Agreement on Different Nonperipheral Washout Patterns

**FIGURE 3:**
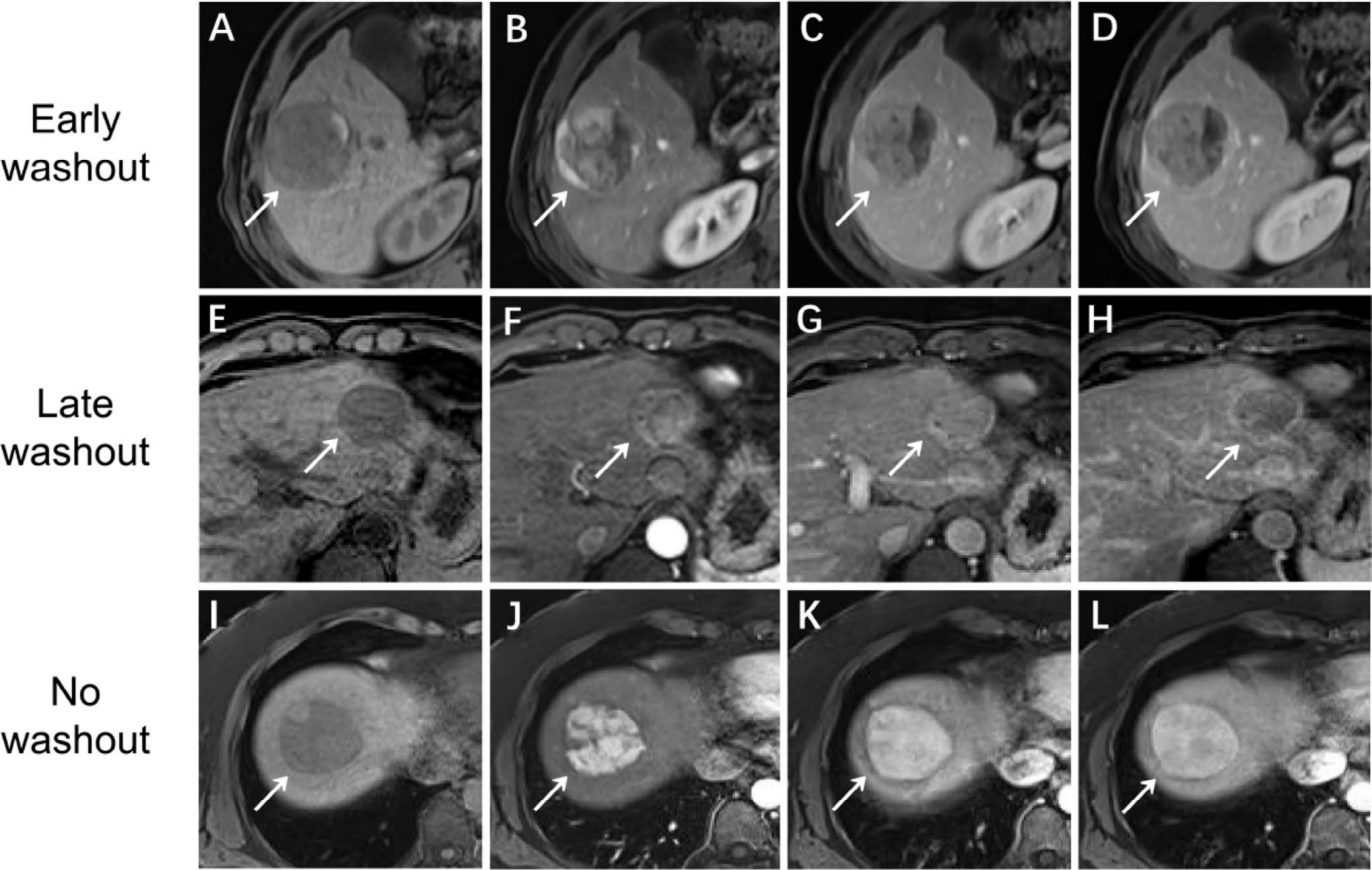
Examples of different nonperipheral washout patterns. (A–D) A 45‐year‐old male with a 5.2‐cm HCC in segment V/VI. The lesion demonstrates hypointensity on axial T1‐weighted pre‐contrast imaging (A), shows nonrim arterial phase hyperenhancement (APHE) (B), and demonstrates washout in both portal venous (C) and delayed phases (D). The patient experienced recurrence 3 months post‐resection. (E–H) A 44‐year‐old male with a 4.0‐cm HCC in segment II/III. The lesion demonstrates hypointensity on axial T1‐weighted pre‐contrast imaging (E) and shows nonrim APHE (F). Washout is absent in the portal venous phase (G) but present in the delayed phase (H). The patient remained recurrence‐free at 39 months post‐resection. (I–L) A 66‐year‐old female with a 4.9‐cm HCC in segment VII/VIII. The lesion demonstrates hypointensity on axial T1‐weighted pre‐contrast imaging (I) and shows nonrim APHE (J). Washout is absent in both the portal venous (K) and delayed phases (L). The patient remained recurrence‐free at 34 months post‐resection.

Based on the ‘observed washout pattern’, 60.1% (367/611), 15.5% (95/611), and 24.4% (149/611) of HCCs showed early, late, and no washout, respectively. Representative patient images for different non‐peripheral washout patterns are shown in Figure 3.

The inter‐reader agreement on nonperipheral washout patterns was substantial for both the synthesized (rounds 1 and 2; weighted *κ*, 0.70) and observed (round 3; weighted *κ*, 0.74) results. The inter‐rater agreement on early washout (present or absent) was substantial, with Fleiss' *κ* of 0.67 for round 1 (when only the pre‐contrast and PVP images were evaluated) and 0.70 for round 3 (when the pre‐contrast, PVP, and DP images were evaluated), respectively. Similarly, the inter‐rater agreement on late washout (present or absent) was also substantial, with Fleiss' *κ* of 0.70 for round 2 (when only the pre‐contrast and DP images were evaluated) and 0.74 for round 3, respectively (Table S3).

The intra‐rater agreement between the synthesized (rounds 1 and 2) and observed results (round 3) was excellent for all readers regarding nonperipheral washout patterns (weighted *κ*, R1, 0.84; R2, 0.82; R3, 0.81), early washout (Cohen's kappa: R1, 0.85; R2, 0.88; R3, 0.86), and late washout (Cohen's kappa: R1, 0.94; R2, 0.84; R3, 0.84) (Table S3).

The inter‐rater agreement on other imaging features was fair to excellent (*k* or ICC, 0.25 to 0.98) (Table S4).

### 3.3 Clinicopathologic Characteristics for Different Nonperipheral Washout Patterns

Clinicopathologic characteristics for different nonperipheral washout patterns are summarized in Table 2.

**TABLE 2:** Differences in clinicopathologic characteristics according to the nonperipheral washout pattern.

Characteristics	No washout (*n* = 149)	Late washout (*n* = 95)	Early washout (*n* = 367)	*p* overall	*p* ^a^ (no vs. late)	*p* ^a^ (no vs. early)	*p* ^a^ (late vs. early)
Patient demographics							
Age (years)	56.0 (48.0, 64.0)	56.0 (49.0, 64.0)	54.0 (46.0, 63.0)	0.21	—	—	—
Sex (Male)	121 (81.2)	82 (86.3)	317 (86.4)	0.31	—	—	—
Chronic liver disease^b^							
Hepatitis B virus	134 (89.9)	82 (86.3)	320 (87.2)	0.62	—	—	—
Hepatitis C virus	2 (1.3)	4 (4.2)	5 (1.4)	0.18	—	—	—
Alcohol use	8 (5.4)	1 (1.1)	18 (4.9)	0.22	—	—	—
MASLD	4 (2.7)	1 (1.1)	8 (2.2)	0.73	—	—	—
Other	0 (0.0)	0 (0.0)	1 (0.3)	> 0.99	—	—	—
Cryptogenic	9 (6.0)	10 (10.5)	27 (7.4)	0.42	—	—	—
ALBI grade							
1	111 (74.5)	73 (76.8)	285 (77.7)	0.74	—	—	—
2	38 (25.5)	22 (23.2)	82 (22.3)				
Tumour burden							
BCLC stage							
0	54 (36.2)	20 (21.1)	46 (12.5)	**< 0.001**	0.06	**< 0.001**	**0.003**
A	95 (63.8)	75 (78.9)	321 (87.5)				
Size of tumour (cm)	2.4 (1.8, 3.0)	2.8 (2.2, 4.0)	3.8 (2.5, 6.0)	**< 0.001**	**0.013**	**< 0.001**	**< 0.001**
Laboratory results							
α‐fetoprotein (> 400 ng/mL)	32 (21.5)	15 (15.8)	100 (27.2)	**0.046**	0.82	0.52	0.06
Histopathologic features							
Cirrhosis	84 (56.4)	56 (58.9)	207 (56.4)	0.90	—	—	—
Edmondson–Steiner grade (III/IV)	38 (25.5)	21 (22.1)	146 (39.8)	**< 0.001**	0.88	**0.003**	**0.002**
Microvascular invasion	34 (22.8)	26 (27.4)	155 (42.2)	**< 0.001**	> 0.99	**0.002**	**0.014**
MTM subtype^c^	7 (7.5)	2 (4.5)	54 (26.0)	**< 0.001**	> 0.99	**< 0.001**	**0.001**
Satellite nodules^d^	5 (4.8)	3 (5.3)	18 (7.2)	0.66	—	—	—
LI‐RADS category^e^							
LR‐4	48 (34.5)	3 (3.4)	3 (0.9)	**< 0.001**	**< 0.001**	**< 0.001**	0.06
LR‐5	85 (61.2)	82 (94.3)	306 (89.7)				
LR‐M	6 (4.3)	2 (2.3)	32 (9.4)				

*Note:* Continuous variables are expressed as medians with IQRs in parentheses, while categorical variables are expressed as numbers with percentages in parentheses. Unless stated otherwise, continuous variables were compared using the Kruskal–Wallis test and categorical variables using the *χ* ^2^ test. Bold *p* values indicate statistically significant differences (*p* < 0.05).

Abbreviations: ALBI = albumin–bilirubin, BCLC = Barcelona Clinic Liver Cancer, M = male, MASLD = metabolic dysfunction–associated steatotic liver disease, MTM = macrotrabecular‐massive.

^a^ For characteristics with significant differences across three groups (*p* < 0.05), pairwise comparisons were performed using *χ* ^2^/Fisher tests (categorical) or Dunn's test (continuous/ordinal), with Bonferroni‐adjusted *p*‐values.

^b^ More than one aetiology could be present for each patient.

^c^ Data available for 345 patients (no washout, *n* = 93; late washout, *n* = 44; early washout, *n* = 208).

^d^ Data available for 411 patients (no washout, *n* = 104; late washout, *n* = 57; early washout, *n* = 250).

^e^ Comparisons were made among patients fulfilling LI‐RADS high‐risk criteria (no washout, *n* = 139; late washout, *n* = 87; early washout, *n* = 341).

Compared to the late washout group, tumours demonstrating early washout were larger (median size: 3.8 cm vs. 2.8 cm; adjusted *p* < 0.001) and showed more frequent aggressive histopathologic features, including the MTM subtype (26.0% vs. 4.5%; adjusted *p* = 0.001), Edmondson–Steiner III/IV grade (39.8% vs. 22.1%; adjusted *p* = 0.002), and MVI (42.2% vs. 27.4%; adjusted *p* = 0.014).

No difference in cirrhosis (*p* = 0.90) or the ALBI grade (*p* = 0.74) was detected among different nonperipheral washout patterns.

### 3.4 Imaging Features for Different Nonperipheral Washout Patterns

Imaging features for different nonperipheral washout patterns are summarized in Table S5.

Compared to the late washout group, tumours showing early washout also demonstrated higher frequencies of the nodule‐in‐nodule architecture (40.1% vs. 25.3%; adjusted *p* = 0.008), mosaic architecture (31.9% vs. 8.4%; adjusted *p* < 0.001), blood products in mass (30.8% vs. 11.6%; adjusted *p* < 0.001), necrosis or severe ischemia (43.1% vs. 22.1%; adjusted *p* < 0.001), corona enhancement (40.6% vs. 22.1%; adjusted *p* = 0.001). T2 peritumoral hyperintensity (25.3% vs. 12.6%; adjusted *p* = 0.008), PVP peritumoral hypoenhancement (26.7% vs. 6.3%; adjusted *p* < 0.001), low ADC value (53.2% vs. 33.7%; adjusted *p* = 0.002), intratumoral artery (31.3% vs. 9.5%; adjusted *p* < 0.001), non‐smooth tumour margin (80.9% vs. 60.0%; adjusted *p* < 0.001), the VICT2 trait (39.5% vs. 17.9%; adjusted *p* < 0.001), and non‐simple nodule type (58.6% vs. 34.7%; adjusted *p* < 0.001). In contrast, complete capsule (49.5% vs. 24.3%; adjusted *p* < 0.001) and arterial phase hyperenhancement proportion > 50% (95.8% vs. 70.6%; adjusted *p* < 0.001) were more frequently observed in the late washout group than the early washout group.

### 3.5 Prognostic Implications of Different Nonperipheral Washout Patterns

During a median follow‐up period of 45.7 months (IQR, 34.5–59.5 months), 238 (39.0%, 238/611) patients experienced recurrence (including 149 early recurrences) and 111 (18.2%, 111/611) died (including 28 deaths within 2 years).

**FIGURE 4:**
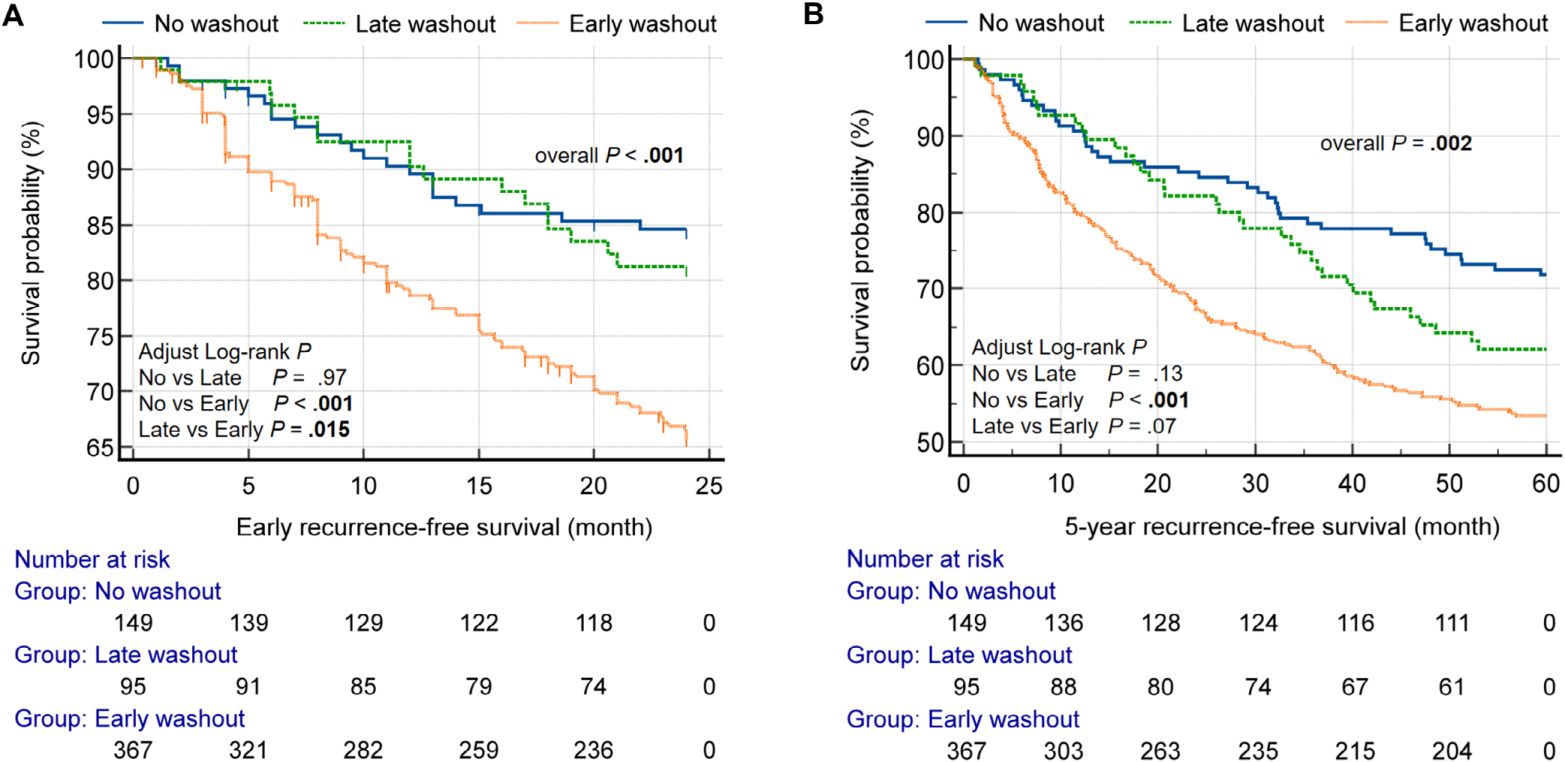
Early (A) and 5‐year (B) recurrence‐free outcomes for different nonperipheral washout patterns.

The median RFS was 50.2 months (95% CI, 40.2–60.6 months) for the early washout group, 64.7 months (95% CI, 46.0 months to NR [not reached]) for the late washout group, and 96.9 months (95% CI, 59.4 months to NR) for the no washout group (*p* < 0.001). The cumulative eRFS rates were 67.3%, 82.1%, and 85.2% for the early, late, and no washout groups, respectively (*p* < 0.001) (Figure 4). The early washout group had significantly worse eRFS and 5‐year RFS than the no washout group (adjusted log‐rank *p* < 0.001 for both), whereas no difference was observed between the late and no washout groups (adjusted log‐rank *p* = 0.97 and 0.13, respectively).

The prognostic value of early washout was compared with that of conventional washout (i.e., washout in either the PVP or DP). While both early and conventional washout were predictive of eRFS and 5‐year RFS in the univariable Cox regression analyses (Table S6), only early washout remained predictive of both eRFS (HR = 1.86; 95% CI, 1.29–2.69; *p* < 0.001) and 5‐year RFS (HR = 1.46; 95% CI, 1.12–1.92; *p* = 0.006) in the multivariable Cox regression analysis after adjusting for other well‐established prognostic factors (Table 3).

**TABLE 3:** Cox regression analyses for early and 5‐year recurrence‐free survival among all patients.

Variable	Early recurrence‐free survival				5‐year recurrence‐free survival			
	Univariable HR	*p*	Multivariable HR	*p*	Univariable HR	*p*	Multivariable HR	*p*
Washout classification^a^								
Early washout (present vs. absent)	2.29 (1.59, 3.28)	**< 0.001**	1.86 (1.29, 2.69)	**< 0.001**	1.69 (1.30, 2.22)	**< 0.001**	1.46 (1.12, 1.92)	**0.006**
Conventional washout (present vs. absent)	2.19 (1.39, 3.42)	**< 0.001**	—	—	1.82 (1.31, 2.54)	**< 0.001**	—	—
Imaging								
At least one LR‐M feature (present vs. absent)	1.63 (1.02, 2.61)	**0.041**	—	—	1.58 (1.07, 2.33)	**0.020**	—	—
Tumour size (cm)	1.14 (1.09, 1.19)	**< 0.001**	—	—	1.11 (1.06, 1.15)	**< 0.001**	—	—
Mosaic architecture (present vs. absent)	1.62 (1.15, 2.28)	**0.006**	—	—	1.45 (1.10, 1.92)	**0.009**	—	—
Blood products in mass (present vs. absent)	1.69 (1.19, 2.40)	**0.003**	—	—	1.71 (1.30, 2.24)	**< 0.001**	—	—
Rim APHE (present vs. absent)	1.60 (0.89, 2.88)	**0.12**	—	—	1.68 (1.06, 2.69)	**0.029**	—	—
Necrosis or severe ischemia (present vs. absent)	1.53 (1.10, 2.14)	**0.010**	—	—	1.63 (1.27, 2.11)	**< 0.001**	—	—
T2WI peritumoral hyperintensity (present vs. absent)	1.62 (1.14, 2.29)	**0.007**	—	—	1.30 (0.97, 1.76)	0.08	—	—
PVP peritumoral hypoenhancement (present vs. absent)	1.81 (1.27, 2.57)	**< 0.001**	—	—	1.43 (1.05, 1.93)	**0.021**	—	—
APHE proportion (> 50% vs. ≤ 50%)	0.65 (0.46, 0.94)	**0.021**	—	—	0.66 (0.50, 0.88)	**0.005**	—	—
Intra‐tumoural artery (present vs. absent)	2.16 (1.55, 3.00)	**< 0.001**	—	—	1.70 (1.29, 2.24)	**< 0.001**	—	—
Complete capsule (absent vs. present)	1.67 (1.11, 2.51)	**0.010**	—	—	1.51 (1.15, 1.99)	**0.003**	—	—
Non‐smooth tumour margin (present vs. absent)	1.93 (1.29, 2.88)	**0.001**	—	—	1.63 (1.20, 2.20)	**0.002**	—	—
The VICT2 trait (present vs. absent)	1.50 (1.09, 2.06)	**0.013**	—	—	1.13 (0.87, 1.48)	0.35	—	—
Tumour growth subtype (non‐simple nodular vs. simple nodular)	2.02 (1.47, 2.78)	**< 0.001**	—	—	1.59 (1.24, 2.04)	**< 0.001**	—	—
Involvement of liver capsule (present vs. absent)	1.40 (1.00, 1.98)	0.05	—	—	1.44 (1.09, 1.89)	**0.01**	—	—
Clinical								
Age (years)	0.98 (0.97, 1.00)	**0.008**	—	—	0.99 (0.98, 1.00)	**0.017**	—	—
Sex (M vs. F)	0.97 (0.63, 1.50)	0.90	—	—	1.01 (0.71, 1.43)	0.95	—	—
ALBI grade (grade 2 vs. grade 1)	1.10 (0.83, 1.46)	0.49	—	—	1.05 (0.78, 1.40)	0.74	—	—
BCLC stage (0 vs. A)	1.32 (0.96, 1.83)	0.09	—	—	1.31 (0.94, 1.82)	0.11	—	—
α‐fetoprotein (ng/mL)^b^	1.18 (1.11, 1.25)	**< 0.001**	1.11 (1.05, 1.18)	**< 0.001**	1.12 (1.07, 1.18)	**< 0.001**	1.08 (1.02, 1.13)	**0.003**
Surgical								
Open surgery (yes vs. no)	1.71 (1.17, 2.50)	**0.005**	—	—	1.48 (1.11, 1.97)	**0.007**	—	—
Major hepatectomy (yes vs. no)	1.11 (0.69, 1.80)	0.66	—	—	0.92 (0.62, 1.39)	0.71	—	—
Histopathologic								
Microvascular invasion (present vs. absent)	3.17 (2.31, 4.35)	**< 0.001**	2.51 (1.81, 3.50)	**< 0.001**	2.63 (2.05, 3.38)	**< 0.001**	2.29 (1.77, 2.97)	**< 0.001**
Edmondson–Steiner grade (III/IV vs. I/II)	1.42 (1.03, 1.95)	0.030	—	—	1.18 (0.91, 1.53)	0.20	—	—

*Note:* Data in parentheses are 95% CIs. After accounting for high collinearity (i.e., known clinical collinearity, such as continuous and categorical forms of laboratory indices, Spearman's rho > 0.6, or a variance inflation factor > 5), 13 features (early washout, tumour size, blood products in mass, PVP peritumoral hypoenhancement, APHE proportion, non‐smooth tumour margin, tumour growth subtype, at least one LR‐M feature, age, α‐fetoprotein, open surgery, microvascular invasion, and Edmondson–Steiner grade) were included in the multivariable Cox regression model to evaluate early recurrence‐free survival and 13 features (early washout, tumour size, blood products in mass, PVP peritumoral hypoenhancement, APHE proportion, non‐smooth tumour margin, tumour growth subtype, at least one LR‐M feature, involvement of liver capsule, age, α‐fetoprotein, open surgery, and microvascular invasion) were included in the multivariable Cox regression model to evaluate 5‐year recurrence‐free survival. Detailed results of the univariable analyses are presented in Table S6. Bold *p* values indicate statistically significant predictors in Cox proportional hazards regression models (*p* < 0.05).

^a^ Early washout was defined as nonperipheral washout observed in the portal venous phase (regardless of the findings in the delayed phase), while conventional washout was defined as nonperipheral washout observed in either the portal venous phase or the delayed phase.

^b^ Data were computed after logarithmic transformation.

In sensitivity analyses limited to patients with satellite‐nodule data (Tables S7 and S8) and to the LI‐RADS high‐risk subgroup (Tables S9 and S10), early washout remained an independent predictor for both eRFS and 5‐year RFS (all *p* < 0.05). Consistently, multivariable subgroup analyses demonstrated that the independent association between early washout and shorter eRFS remained robust across nearly all clinical and radiological strata (Table 4). Notably, no significant effect modification was observed (all interaction *p* > 0.05). Similar consistency in the prognostic value of early washout was observed for 5‐year RFS, with detailed multivariable results provided in Table S11.

**TABLE 4:** Impact of early washout on early recurrence‐free survival according to different subgroups.

Subgroup	No. of events (%)^a^	Univariable analysis			Multivariable analysis adjusted for the other predictive variables			
		HR (95% CI)	*p*	*p* for interaction	HR (95% CI)	*p*	*p* for interaction	Variables adjusted for
AFP								
α‐fetoprotein ≤ 400 ng/mL	74 (27.7)	2.05 (1.34–3.15)	**0.001**	0.55	1.90 (1.24–2.93)	**0.003**	> 0.99	MVI
α‐fetoprotein > 400 ng/mL	46 (46.0)	2.54 (1.28–5.03)	**0.008**		1.86 (0.91–3.79)	0.09		MVI
Tumour size								
Tumour size ≤ 2 cm	13 (31.0)	3.76 (1.50–9.43)	**0.005**	0.18	3.88 (1.53–9.85)	**0.004**	0.08	MVI, LnAFP
Tumour size > 2 cm	107 (32.9)	1.91 (1.29–2.84)	**0.001**		1.57 (1.05–2.34)	**0.03**		MVI, LnAFP
Microvascular invasion								
Absent	41 (19.3)	1.55 (0.94–2.57)	0.09	0.22	1.59 (0.96–2.64)	0.07	0.34	LnAFP
Present	79 (51.0)	2.48 (1.42–4.30)	**0.001**		2.35 (1.34–4.10)	**0.003**		LnAFP
Edmondson–Steiner grade								
I/II	64 (29.0)	1.88 (1.23–2.89)	**0.004**	0.18	1.64 (1.06–2.54)	**0.03**	0.22	MVI, LnAFP
III/IV	56 (38.4)	3.26 (1.56–6.85)	**0.002**		2.57 (1.21–5.46)	**0.01**		MVI, LnAFP
Cirrhosis								
Absent	40 (27.6)	2.60 (1.34–5.08)	**0.005**	0.66	1.96 (0.98–3.92)	0.06	0.99	MVI, LnAFP
Present	80 (36.0)	2.18 (1.42–3.35)	**< 0.001**		1.93 (1.25–2.99)	**0.003**		MVI, LnAFP
Liver steatosis								
Absent	98 (32.8)	2.29 (1.53–3.41)	**< 0.001**	0.98	1.86 (1.24–2.79)	**0.003**	0.99	MVI, LnAFP
Present	22 (32.4)	2.30 (0.98–5.39)	0.06		1.86 (0.79–4.39)	0.16		MVI, LnAFP
Enhancing capsule								
Present	119 (32.9)	2.34 (1.59–3.42)	**< 0.001**	0.54	1.87 (1.27–2.77)	**0.002**	0.72	MVI, LnAFP
MRI field strength								
1.5 T	74 (38.9)	2.38 (1.49–3.80)	**0.009**	0.75	1.86 (1.15–2.99)	**0.01**	0.82	MVI, LnAFP
3.0 T	46 (26.0)	2.14 (1.21–3.78)	**< 0.001**		1.83 (1.02–3.26)	**0.04**		MVI, LnAFP
Resection extent								
Minor resection	104 (33.2)	2.37 (1.62–3.46)	**< 0.001**	0.53	2.02 (1.38–2.97)	**< 0.001**	0.25	MVI, LnAFP
Resection type								
Laparoscopic surgery	26 (24.8)	2.69 (1.22–5.95)	**< 0.001**	0.60	2.38 (1.07–5.29)	**0.03**	0.43	MVI, LnAFP
Open surgery	94 (35.9)	2.14 (1.42–3.21)	0.01		1.72 (1.14–2.61)	**0.01**		MVI, LnAFP
Resection margin								
Resection margin < 1 cm	19 (26.4)	4.36 (1.48–12.82)	**0.007**	0.15	3.65 (1.23–10.83)	**0.02**	0.21	MVI, LnAFP
Resection margin ≥ 1 cm	43 (32.1)	1.79 (1.01–3.17)	0.05		1.74 (0.97–3.09)	0.06		MVI, LnAFP

*Note:* Data in parentheses are 95% CIs. Bold *p* values indicate statistically significant predictors in Cox proportional hazards regression models (*p* < 0.05).

^a^ No. of events (%) in patients with early washout.

In the Fine–Gray competing‐risk models, which accounted for non‐recurrence‐related deaths, early washout remained an independent predictor for both early recurrence (subdistribution HR [sHR] = 1.92; 95% CI, 1.31–2.81; *p* < 0.001) and 5‐year recurrence (sHR = 1.48; 95% CI, 1.12–1.96; *p* = 0.007). Full model specifications and coefficients are summarized in Tables S12 and S13.

### 3.6 Factors Impacting the Interpretations of Different Nonperipheral Washout Patterns

The impacts of MRI field strength, cirrhosis, liver steatosis, enhancing capsule, and mosaic architecture on the interpretation of different nonperipheral washout patterns are summarized in Table S14.

Briefly, conventional washout was more frequently observed on 1.5 T MRI (80.0% vs. 71.1%, *p* = 0.01), in the absence of liver steatosis (77.3% vs. 68.1%, *p* = 0.04), as well as in the presence of enhancing capsule (78.4% vs. 27.3%, *p* < 0.001) and the mosaic architecture (91.9% vs. 71.0%, *p* < 0.001). Similarly, early washout was more frequently observed in tumours with an enhancing capsule (62.6% vs. 15.2%, *p* < 0.001) and a mosaic architecture (86.0% vs. 52.6%, *p* < 0.001). However, no difference in the frequency of early washout was detected across different MRI field strengths or cirrhosis and steatosis status (*p*, 0.53 to 0.98).

## 4 Discussion

The timing of nonperipheral washout carries distinct prognostic implications. In this multicentre retrospective cohort study of 611 patients who underwent curative resection for single BCLC 0/A HCC, we found that early washout (identified in the PVP) demonstrated stronger prognostic value than the conventional washout definition and was associated with poorer eRFS and 5‐year RFS. This association remained significant even after adjustment for established clinicopathological and imaging prognostic factors in the multivariable Cox regression analysis.

The prognostic significance of nonperipheral washout has been inconsistently reported across studies [13, 27, 28, 29]. While some reports indicated a strong correlation between nonperipheral washout and adverse outcomes [13, 27, 28], others described a negligible role [29]. This heterogeneity may be partly attributed to a lack of distinction among temporal patterns, as prior studies often treated nonperipheral washout in the PVP and DP as equivalent imaging markers. Such a ‘one‐size‐fits‐all’ approach may have obscured high‐risk subgroups and conflated biologically distinct tumours, thereby contributing to contradictory findings in the literature [29]. In contrast, HCCs showing early washout demonstrated more frequent aggressive imaging (e.g., less frequent complete capsule formation and more prevalent necrosis or severe ischemia) and histoprognostic features (higher Edmondson–Steiner grade, greater frequency of MVI, and MTM subtype). Furthermore, early washout also demonstrated stronger prognostic value in comparison to conventional washout. Notably, early washout remained a robust independent predictor of worse eRFS and 5‐year RFS, even after adjustment for widely acknowledged clinicopathologic prognostic factors. Collectively, these results highlight that, by distinguishing HCCs with different temporal patterns of nonperipheral washout, early washout may serve as a more specific prognostic biomarker, allowing better prediction of early recurrence risk than the conventional definition of washout, which treats the PVP and DP as equivalent.

Notably, our study proposes an ECA‐MRI approach to characterize the temporal evolution of nonperipheral washout. Although gadoxetate disodium (EOB)‐enhanced MRI is widely used for preoperative HCC assessment, ECA‐MRI remains preferred when transient severe motion artefacts are a concern, robust arterial‐phase imaging is prioritized, or hepatobiliary‐phase quality may be compromised (e.g., advanced cirrhosis, biliary obstruction). In this context, early washout is potentially extrapolatable to EOB‐MRI since PVP enhancement is predominantly driven by extracellular distribution of the contrast agent [30]. This extrapolation is supported by Tomino et al., who reported that a PVP tumour‐to‐liver signal intensity ratio of ≤ 0.68 on EOB‐MRI was associated with worse overall survival in patients who received resection for HCC [31]. However, late washout may not be applied to the transitional or hepatobiliary phase, because hepatocyte uptake could cause apparent hypointensity or pseudo‐washout that obscures the detection of “true” washout.

Quantitative measurement of signal intensity is influenced by scanning parameters and requires region‐of‐interest placement, which may be operator dependent and lacks standardized threshold criteria [9, 32]. In our work, we adopted a more robust qualitative approach for the temporal pattern of nonperipheral washout. While visual assessment may introduce inherent subjectivity [15], our study achieved substantial inter‐rater agreement in classifying the temporal washout pattern (weighted kappa, 0.70–0.74). Notably, using a three‐round blinded image‐analysis protocol, we found that different approaches to assessing temporal washout patterns, whether independently synthesized or simultaneously evaluated, consistently exhibited reproducible inter‐ and intra‐rater agreement. However, agreement may be modestly lower for highly distinctive lesions, such as very large tumours or those with characteristic mosaic architecture. Subgroup analyses further confirmed the robust prognostic value of early washout, which remained significant across nearly all subgroups (e.g., by tumour size, Edmondson–Steiner grade, cirrhosis status, and MRI field strength). These results may position washout timing as a robust, readily accessible biomarker that can be readily applied in routine practice.

The differential prognostic impact of early versus late washout likely reflects fundamental differences in tumour hemodynamics and vascular architecture [10, 16]. The PVP represents a period of rapidly evolving hepatic hemodynamics following peak arterial enhancement, whereas the DP corresponds to a relatively stable equilibrium state [9]. Therefore, early washout may reflect accelerated blood flow through the tumour with minimal sinusoidal retention, a hemodynamic pattern characteristic of abundant arteriovenous shunting and disorganized microvasculature [16]. This phenomenon aligns with the findings from Wu et al., who found that early washout (< 60 s) on contrast‐enhanced ultrasound was a strong predictor of the MTM subtype of HCC (OR = 8.82) and independently predicted worse overall survival (HR = 2.26) [33]. Although contrast‐enhanced ultrasound uses blood pool agents [34], which differ from ECA, the consistent association between early washout and aggressive tumour behaviour across imaging modalities underscores that rapid contrast clearance may fundamentally reflect an aggressive tumour biology characterized by aberrant vascularization and primitive microvasculature [35]. Interestingly, HCCs with late washout exhibited histopathologic characteristics and a prognosis similar to those without washout. However, given the relatively small numbers of the late washout (*n* = 95, 15.5%) and no washout (*n* = 149, 24.4%) groups, our study may have been underpowered to detect subtle prognostic differences between these two groups. Therefore, larger studies with enriched late‐washout cohorts are warranted to confirm whether these two washout patterns are biologically similar.

Several limitations should be noted. First, the retrospective design might have introduced selection biases and heterogeneity in scanning protocols across centres. However, standardized LI‐RADS compliance and rigorous image analysis may mitigate these concerns. Second, the HBV‐predominant aetiology (87.7%) may limit the generalizability of our findings to non‐viral HCCs, including those related to metabolic dysfunction–associated steatotic liver disease or other aetiologies, which deserve dedicated validation. Third, the study population was restricted to patients with single BCLC stage 0 or A HCC who underwent curative resection, which may limit applicability to patients with greater tumour burden or different treatment pathways. However, this focused design provided a homogeneous cohort for evaluating the independent prognostic value of washout timing while reducing confounding from tumour and treatment heterogeneity. Fourthly, residual recall bias for distinctive lesions may not be fully excluded, although outcome analyses were based on the consensus assessment from the integrated reading round. Finally, although PVP‐based early washout may be conceptually explored on EOB‐MRI, the full early‐versus‐late washout classification was developed using ECA‐MRI and cannot be directly extrapolated to EOB‐MRI. Future prospective studies with multi‐aetiology cohorts, broader tumour stages, and diverse treatment modalities are warranted to validate the prognostic role of washout timing and to explore its biologic basis and therapeutic implications.

In conclusion, in patients undergoing curative resection for BCLC 0/A single HCC, early washout identified on the PVP of ECA‐MRI is an independent risk factor for both early and 5‐year recurrence. This readily accessible imaging marker may facilitate risk‐stratified treatment.

## Author Contributions

Guarantors of integrity of entire study, Yanshu Wang, Hanyu Jiang and Bin Song; study concepts/study design or data acquisition or data analysis/interpretation, all authors; manuscript drafting or manuscript revision for important intellectual content, all authors; approval of final version of submitted manuscript, all authors; agrees to ensure any questions related to the work are appropriately resolved, all authors; literature research, Yingyi Wu, Junhan Pan, Ting Yang, Yanshu Wang, Hanyu Jiang; clinical studies, all authors; statistical analysis, Yingyi Wu, Junhan Pan, Ting Yang, Yanshu Wang, Hanyu Jiang; and manuscript editing, all authors.

## Funding

This work was supported by National Key Research and Development Program of China (Grant No. 2023YFC2414202), National Natural Science Foundation of China (Grant No. 82471960, U22A20343) and Natural Science Foundation of Sichuan Province (Grant No. 2024ZDZX0017, 2024NSFSC1799).

## Conflicts of Interest

All authors disclose no conflicts of interest relevant to this work. Hanyu Jiang is a stock owner of Kanghong Technology Co. Ltd. Maxime Ronot received educational fees from Sirtex, Guerbet, Ipsen, Servier, GE Healthcare, Angiodynamics, and consulting fees (paid to the institution) from Quantum Surgical. Hui‐chuan Sun received speaker fees from AstraZeneca, BeiGene, Eisai, Hengrui, Innovent, MSD, Qilu Pharmaceutical, Roche, and Zelgen; and research grants from Sino Biopharmaceutical, Eisai, Innovent, Roche, and MSD.

## Supporting information


**Appendix S1:** Imaging acquisition details.
**Appendix S2:** Nonperipheral washout assessment details.
**Figure S1:** Nonperipheral washout assessment flowchart.
**Appendix S3:** Statistical analysis details.
**Table S1:** MR sequences and parameters.
**Table S2:** Definitions of imaging features.
**Table S3:** Inter‐ and intra‐rater agreement in nonperipheral washout pattern.
**Table S4:** Inter‐rater agreement for all evaluated imaging features.
**Table S5:** Differences in imaging features according to nonperipheral washout pattern.
**Table S6:** Univariable Cox regression analyses for early and 5‐year recurrence‐free survival among all patients.
**Table S7:** Cox regression analyses for early recurrence‐free survival among patients with available satellite nodule data.
**Table S8:** Cox regression analyses for 5‐year recurrence‐free survival among patients with available satellite nodule data.
**Table S9:** Cox regression analyses for early recurrence‐free survival in LI‐RADS high‐risk population.
**Table S10:** Cox regression analyses for 5‐year recurrence‐free survival in LI‐RADS high‐risk population.
**Table S11:** Impact of early washout on 5‐year recurrence‐free survival according to different subgroups.
**Table S12:** Uni‐ and multi‐variable Fine–Gray competing risks analyses for time to early recurrence.
**Table S13:** Uni‐ and multi‐variable Fine–Gray competing risks analyses for time to 5‐year recurrence.
**Table S14:** Frequencies of different nonperipheral washout patterns according to clinical and imaging factors that may impact image interpretations.

**Supplementary Appendix 2:** Completed STROBE checklist.

## Data Availability

The data supporting the findings of this study are not publicly available due to privacy restrictions but are available from the corresponding author upon reasonable request.
