# Supplementary material for: Temporal Differentiation of Nonperipheral Washout Refines Prognostic Stratification in HCC
Source: Liver Int. 2026 Aug 25;46(9):e70838. doi: 10.1111/liv.70838 (PMC13507576; doi:10.1111/liv.70838)
Supplement: Supplementary file 2 [file LIV-46-0-s001.docx]

**Temporal Differentiation of Nonperipheral Washout Refines Prognostic Stratification in HCC**

Supplementary Appendix 1：Additional methodological details, supplementary tables and figures

[Appendix S1: Imaging acquisition details	3](#_Toc17925)

[Appendix S2. Nonperipheral washout assessment details	4](#_Toc26520)

[Figure S1. Nonperipheral washout assessment flowchart.	5](#_Toc5865)

[Appendix S3: Statistical analysis details	6](#_Toc18564)

[Table S1. MR sequences and parameters	9](#_Toc16888)

[Table S2. Definitions of imaging features	12](#_Toc1022)

[Table S3. Inter- and intra-rater agreement in nonperipheral washout pattern	18](#_Toc26421)

[Table S4. Inter-rater agreement for all evaluated imaging features	19](#_Toc25702)

[Table S5. Differences in imaging features according to nonperipheral washout pattern	21](#_Toc20548)

[Table S6. Univariable Cox regression analyses for early and 5-year recurrence-free survival among all patients	23](#_Toc6792)

[Table S7. Cox regression analyses for early recurrence-free survival among patients with available satellite nodule data	25](#_Toc10639)

[Table S8. Cox regression analyses for 5-year recurrence-free survival among patients with available satellite nodule data	26](#_Toc11444)

[Table S9. Cox regression analyses for early recurrence-free survival in LI-RADS high-risk population	27](#_Toc25997)

[Table S10. Cox regression analyses for 5-year recurrence-free survival in LI-RADS high-risk population	28](#_Toc15193)

[Table S11. Impact of early washout on 5-year recurrence-free survival according to different subgroups	29](#_Toc9052)

[Table S12. Uni- and multivariable Fine-Gray competing risks analyses for time to early recurrence	30](#_Toc12234)

[Table S13. Uni- and multivariable Fine-Gray competing risks analyses for time to 5-year recurrence	32](#_Toc31500)

[Table S14. Frequencies of different nonperipheral washout patterns according to clinical and imaging factors that may impact image interpretations	35](#_Toc19925)

**Appendix S1: Imaging acquisition details**

All contrast-enhanced MRI examinations were performed on one of nine 1.5- or 3.0-T scanners (Siemens MAGNETOM Skyra 3.0 T; Siemens TrioTim 3.0 T; Siemens Avanto 1.5 T; GE SIGNA™ Architect 3.0 T; GE SIGNA™ Premier 3.0 T; GE Discovery MR 750 3.0 T; GE Signa HDxt 3.0 T; Philips Ingenia Elition X 3.0 T; uMR588 1.5 T), using extracellular contrast agents (ECAs). For ECA-enhanced MRI, a dose of 0.1 mmol/kg gadopentetate dimeglumine (Magnevist®; Bayer Schering Pharma, Berlin, Germany), gadoterate meglumine (Dotarem®; Guerbet, Paris, France), or gadobenate dimeglumine (MultiHance®; Bracco, Shanghai, China) was administered intravenously at 2.5 mL/s. Patients fasted for 6–8 hours prior to imaging.

The MRI protocol comprised: (a) T2-weighted imaging; (b) diffusion-weighted imaging with system-specific b-value schemes (range, 0–1200 s/mm²) and monoexponential apparent diffusion coefficient maps; (c) in-phase and out-of-phase T1-weighted imaging; and (d) dynamic contrast-enhanced T1-weighted imaging, including pre-contrast, late arterial, portal venous (60 s post-injection), and delayed phases (3 min post-injection). Arterial phase acquisition was performed using either bolus tracking (triggered 7 s after contrast arrival in the celiac trunk) or a multiple-arterial-phase technique (18-s breath-hold starting 20 s after injection, reconstructed at 3-s temporal resolution).

Detailed acquisition parameters for all scanners and sequences are provided in **Table S1**.

**Appendix S2. Nonperipheral washout assessment details**

Prior to the formal readings, all readers underwent a hands-on training with 20 non-study HCC cases to harmonize interpretations. Nonperipheral washout was defined per LI-RADS version 2018 as a reduction in enhancement (in whole or in part) from earlier to later phase, resulting in hypoenhancement relative to liver parenchyma. Of note, the presence of rim arterial phase hyperenhancement (APHE) did not preclude this assessment; the temporal onset of washout was evaluated regardless of the arterial enhancement morphology, provided the washout itself was nonperipheral.

The temporal pattern of nonperipheral washout was classified into three mutually exclusive categories: early washout (unequivocal washout in the portal venous phase [PVP], regardless of the delayed phase [DP]); late washout (unequivocal washout observed only in the DP and not in the PVP); and no washout (absence of washout in the PVP and DP).

To minimize recall bias and inter-phase dependency, a three-round reading scheme was implemented, with at least 4-week intervals between any two rounds. Image sets were anonymized and randomized before each round, and the randomization sequence was generated by (S.Y.W., 5 years of experience). In the first round, readers evaluated the precontrast, AP, and PVP images to determine the presence or absence of nonperipheral washout. In the second round, the same assessment was performed using the precontrast, AP, and DP images. In the third round, readers reviewed the precontrast, AP, PVP, and DP images together to make a comprehensive evaluation.

After completing the first two rounds, each reviewer synthesized their phase-specific assessments to determine a temporal pattern of nonperipheral washout (early, late, or no washout), referred to hereafter as the “synthesized washout pattern”. Following the third round, reviewers assigned the temporal pattern of nonperipheral washout based solely on the third-round evaluation, referred to hereafter as the “observed washout pattern” (illustrated in **Figure S1**).

**Figure S1. Nonperipheral washout assessment flowchart.**

*Abbreviations: AP = arterial phase, PVP = portal venous phase, DP = delayed phase.*

**Appendix S3: Statistical analysis details**

Differences were compared using the Kruskal-Wallis test for non-normally distributed continuous/ordinal variables, and using the chi-square test or Fisher's exact test for categorical variables, as appropriate. For characteristics with significant differences across three groups, post hoc pairwise comparisons were performed with the Bonferroni correction for categorical variables and the Dunn test for continuous or ordinal variables.

***Assessment of Inter- and Intra-reader Agreement***

Agreement for continuous variables (e.g., tumor size) was assessed using the intraclass correlation coefficient (ICC). For categorical imaging features, agreement was assessed using kappa statistics: weighted kappa for multi-category features and Fleiss' kappa (for inter-reader) or Cohen's kappa (for intra-reader) for binary features. In accordance with standard LI-RADS criteria, the LI-RADS high-risk population was defined as patients with cirrhosis, chronic hepatitis B virus (HBV) infection, or a prior history of hepatocellular carcinoma. Consequently, LI-RADS categories were reported exclusively for patients fulfilling these high-risk criteria.

Agreement was considered poor (ICC or κ < 0), slight (ICC or κ, 0-0.20), fair (ICC or κ, 0.20-0.40), moderate (ICC or κ, 0.40-0.60), substantial (ICC or κ, 0.60-0.80), or excellent (ICC or κ> 0.80).

Specific agreement for washout patterns was analyzed as follows:

*Trichotomous washout pattern (early/late/no)*

Inter-reader: Weighted κ (for synthesized [Rounds 1–2] and observed [Round 3] patterns);

Intra-reader: Weighted Cohen’s κ (synthesized vs. observed patterns).

*Binary washout classification (present/absent*)

Intra-reader: Cohen’s κ (early washout: Round 1 vs 3; late washout: Round 2 vs 3);

Inter-reader: Fleiss’ κ (early in Round 1, late in Round 2, both in Round 3).

***Survival analysis***

Early Recurrence-free survival (eRFS) and 5-year RFS were estimated using the Kaplan-Meier method and compared with the log-rank test. Univariable and multivariable Cox proportional hazards regression analyses were conducted to identify predictors of eRFS and 5-year RFS. The proportionality of hazards assumption was verified using Schoenfeld residual analysis. Specifically, predictive variables were first identified by univariable Cox proportional hazards regression analyses, and those with *P*<.05 were assessed for collinearity by Spearman’s correlation analysis and the variance inflation factor. In cases of severe collinearity (i.e., known clinical collinearity, Spearman’s rho>0.6 or the variance inflation factor>5), the strongest predictor was selected for further multivariable analyses. The final multivariable model was built using a backward stepwise selection procedure. No formal a priori sample size calculation was performed for the multivariable Cox regression analysis, given satisfaction of the “rule of thumb” and thus sufficient power.

***Subgroup analyses***

Subgroup analyses were performed to examine the prognostic association between early washout (present vs. absent) and both eRFS and 5-year RFS across strata defined by α-fetoprotein level (≤400 vs. >400 ng/mL), MRI field strength (1.5 T vs. 3.0 T), tumor size, cirrhosis (absent vs. present), liver steatosis (absent vs. present), enhancing capsule status (absent vs. present), Edmondson-Steiner grade (I/II vs. III/IV), microvascular invasion status (absent vs. present), as well as resection margin width (≥1 cm vs. <1 cm), extent (minor vs. major), and type (laparoscopic vs. open). Subgroups with fewer than five events were excluded from the analysis. To avoid overfitting in smaller strata, a parsimonious set of robust confounders retained in the main model was used for adjustment where event counts permitted. Interaction terms were tested to formally assess effect modification.

***Assessment of Factors Impacting the Interpretation of Nonperipheral Washout Patterns***

To assess whether the classification of nonperipheral washout patterns was influenced by specific technical or imaging features, we compared the detection rates of different washout patterns (detailed below) across several subgroups. Specifically, three washout classifications were evaluated: (1) Trichotomous washout pattern (early/late/no); (2) conventional washout (present or absent, defined as washout in either the PVP or DP); and (3) early washout (present or absent, defined as washout specifically detected in the PVP). The distribution of each washout classification was compared across the following factors: liver background (presence of cirrhosis or steatosis on imaging), MRI field strength (1.5 T vs 3.0 T), and two key imaging features that have been previously reported to affect the visualization of nonperipheral washout (i.e., enhancing capsule and mosaic architecture) (1-3).

All the statistical analyses were conducted using R (version 4.4.1; R Foundation for Statistical Computing) or MedCalc (version 20.112; MedCalc Software). A two-tailed *P* < 0.05 was considered statistically significant.

**References:**

1. Min JH, Kang TW, Kim YY, et al. Vanishing washout of hepatocellular carcinoma according to the presence of hepatic steatosis: diagnostic performance of CT and MRI. Eur Radiol 2021;31(5):3315–3325.
2. Hwang JA, Min JH, Kang TW, et al. Assessment of factors affecting washout appearance of hepatocellular carcinoma on CT. Eur Radiol 2021;31(10):7760–7770.
3. Sakamoto K, Tanaka S, Sato K, et al. What is the "washout" of hepatocellular carcinoma as observed on the equilibrium phase CT?: consideration based on the concept of extracellular volume fraction. Jpn J Radiol 2022;40(11):1148–1155.

**Table S1. MR sequences and parameters**

| **Sequence** | **Fat suppression** | **TR (ms)** | **TE (ms)** | **Flip angle (°)** | **ST (mm)** | **Spacing (mm)** | **Matrix size** | **FOV (mm²)** | **Acquisition time (s)** |
| --- | --- | --- | --- | --- | --- | --- | --- | --- | --- |
| **Siemens MAGNETOM Skyra 3.0 T (18-channel body array coil)** | | | | | | | | | |
| T2-weighted 2D FSE | Yes | 2160 | 100 | 160 | 6 | 1.8 | 320x298 | 433x433 | 36 |
| Diffusion-weighted imaging* | Yes | 5600 | 68 | 60 | 6 | 1.6 | 150x276 | 300x289 | 233 |
| In- and out-of-phase T1-weighted imaging | No | 81 | 2.77/4 | 70 | 6 | 1.8 | 352x256 | 400x296 | 24 |
| Dynamic T1-weighted 3D GRE | Yes | 3.55 | 1.92 | 9 | 2.5 | - | 352x256 | 400x296 | 14 |
| **Siemens TrioTim 3.0 T (8-channel body anterior coil)** | | | | | | | | | |
| T2-weighted 2D FSE | Yes | 2700 | 95 | 140 | 6 | 7.8 | 320x147 | 442x254 | Respiratory gating |
| Diffusion-weighted imaging* | Yes | 5900 | 76 | 90 | 6 | 7.8 | 192x154 | 393x383 | 245 |
| In- and out-of-phase T1-weighted imaging | No | 181 | 2.20/3.57 | 65 | 6 | 7.8 | 256x131 | 410x269 | 18 |
| Dynamic T1-weighted 3D GRE | Yes | 3.47 | 1.25 | 9 | 2.4 | - | 320x133 | 434x257 | 17 |
| **GE SIGNA™ Architect 3.0 T (30-channel body anterior coil)** | | | | | | | | | |
| T2-weighted 2D FSE | Yes | 2400 | 85 | 111 | 7 | 2 | 320x192 | 380x304 | 34 |
| Diffusion-weighted imaging* | Yes | 5000 | Minimum | 90 | 7 | 2 | 160x128 | 380x342 | Respiratory gating |
| In- and out-of-phase T1-weighted imaging | No | 233.8 | 2.3/1.1 | 56 | 7 | 2 | 190x288 | 380x343 | 16 |
| Dynamic T1-weighted 3D GRE | Yes | 3.9 | 1.7 | 15 | 3 | - | 320x240 | 380x360 | 15 |
| **GE SIGNA™ Premier 3.0 T (30-channel body anterior coil)** | | | | | | | | | |
| T2-weighted 2D FSE | Yes | 2200 | 85 | 111 | 7 | 2 | 320x204 | 340x380 | 47 |
| Diffusion-weighted imaging* | Yes | 5000 | Minimum | 90 | 7 | 2 | 210x240 | 380x360 | Respiratory gating |
| In- and out-of-phase T1-weighted imaging | No | 146.8 | 2.3/1.1 | 56 | 7 | 2 | 320x192 | 342x380 | 16 |
| Dynamic T1-weighted 3D GRE | Yes | 3.2 | 1.4 | 15 | 2.4 | - | 320x240 | 380x380 | 15 |
| **GE Discovery MR 750 3.0 T (16-channel phased-array torso coil)** | | | | | | | | | |
| T2-weighted 2D FSE | Yes | 6315 | 78 | 111 | 6 | 2 | 288x244 | 360x290 | Respiratory gating |
| Diffusion-weighted imaging* | Yes | 9230 | Minimum | 90 | 6 | 2 | 129x178 | 360x280 | Respiratory gating |
| In- and out-of-phase T1-weighted imaging | No | 150 | 2.6/1.3 | 70 | 6 | 2 | 288x192 | 420x420 | 31 |
| Dynamic T1-weighted 3D GRE | Yes | 4.1 | 1.9 | 15 | 2 | - | 512x512 | 380x330 | 15 |
| **Philips Ingenia Elition X 3.0 T (16-channel body anterior coil)** | | | | | | | | | |
| T2-weighted 2D FSE | Yes | 1883.51 | 90 | 60 | 6.8 | 8.5 | 272x78 | 299x69 | 46 |
| Diffusion-weighted imaging* | Yes | 1683.86 | 80 | 60 | 7 | 8.5 | 142x149 | 141x139 | 52 |
| In- and out-of-phase T1-weighted imaging | No | 164.53 | 2.3/1.1 | 50 | 6 | 7.5 | 256x207 | 206x162 | 11 |
| Dynamic T1-weighted 3D GRE | Yes | 4.2 | 0 | 10 | 3 | 1.5 | 344x252 | 303x222 | 13 |
| **GE Signa HDxt 3.0T (8-channel body anterior coil)** | | | | | | | | | |
| T2-weighted 2D FSE | Yes | 6315 | 88 | 120 | 6 | 0.5 | 320x256 | 380x380 | 150 |
| Diffusion-weighted imaging* | Yes | 10588 | 60 | - | 6 | 0.5 | 154x112 | 380x380 | 107 |
| In- and out-of-phase T1-weighted imaging | No | 4 | 2.3/1.1 | 8 | 5 | - | 288x180 | 380x380 | 16 |
| Dynamic T1-weighted 3D GRE | Yes | 3.2 | 1.5 | 12 | 5 | - | 320x256 | 380x380 | 39 |
| **Siemens Avanto 1.5 T (30-channel body anterior coil)** | | | | | | | | | |
| T2-weighted 2D FSE | Yes | 2530 | 84 | 150 | 6 | 7.8 | 256x187 | 259x251 | 47 |
| Diffusion-weighted imaging* | Yes | 3600 | 88 | 90 | 6 | 7.8 | 192x115 | 310x232 | 92 |
| In- and out-of-phase T1-weighted imaging | No | 72 | 4.92/2.2 | 70 | 6 | 7.8 | 256x158 | 339x225 | 16 |
| Dynamic T1-weighted 3D GRE | Yes | 5.41 | 2.39 | 10 | 2.5 | - | 320x138 | 382x238 | 15 |
| **Siemens Avanto 1.5 T (8-channel body anterior coil)** | | | | | | | | | |
| T2-weighted 2D FSE | Yes | 2710 | 84 | 150 | 7.5 | 8.75 | 256x177 | 308x390 | 27 |
| Diffusion-weighted imaging* | Yes | 2000 | 72 | 70 | 7.5 | 8.75 | 192x125 | 308x379 | 20 |
| In- and out-of-phase T1-weighted imaging | No | 87 | 4.92/2.2 | 70 | 7.5 | 8.75 | 256x187 | 309x390 | 33 |
| Dynamic T1-weighted 3D GRE | Yes | 5.4 | 2.38 | 10 | 2 | - | 320x131 | 241x407 | 15 |
| **uMR588 1.5 T (6-channel body anterior coil)** | | | | | | | | | |
| T2-weighted 2D FSE | Yes | 2600 | 99.2 | 90 | 6.5 | 1.5 | 256x168 | 427x320 | 39 |
| Diffusion-weighted imaging* | Yes | 3350 | 77 | 90 | 6.5 | 10 | 128x92 | 320x400 | Respiratory gating |
| In- and out-of-phase T1-weighted imaging | No | 117.6 | 4.68/2.26 | 60 | 6.5 | 1.3 | 256x174 | 320x400 | 29 |
| Dynamic T1-weighted 3D GRE | Yes | 4.2 | 1.88 | 10 | 2.5 | - | 256x154 | 255x400 | 13 |

Note.— TR = repetition time; TE = echo time; ST = section thickness; FOV = field of view; 2D = two-dimensional; 3D = three-dimensional; FSE = fast spin echo; GRE = gradient recall echo. * Images were acquired under free breath.

**Table S2. Definitions of imaging features**

| **Imaging features** | **Definition** | **MRI sequence** |
| --- | --- | --- |
| **LI-RADS major features** | | |
| Nonrim arterial phase hyperenhancement (APHE) (present vs. absent) | Nonrim-like enhancement of the tumor in the arterial phase unequivocally greater in whole or in part than the liver. (1,2) | Late arterial phase |
| Nonperipheral washout (present vs. absent) | Nonperipheral visually assessed temporal reduction in the enhancement of the tumor in whole or in part relative to composite liver tissue in the portal venous phase or delayed phase. (1,2) | Portal venous or delayed phase for extracellular contrast agents or gadobenate; portal venous phase for gadoxetate |
| Enhancing capsule (present vs. absent) | Smooth, uniform, sharp border around most or all of a tumor, unequivocally thicker or more conspicuous than fibrotic tissue around background nodules, and visible as enhancing rim in portal venous phase, delayed phase, or transitional phase. (1,2) | Portal venous, delayed, or transitional phase |
| Tumor size (cm) | Largest outer-edge-to-outer-edge dimension of a tumor. (1,2) | Pick the phase, sequence, plane in which the margins are clearest. Do not measure in arterial phase or DWI. |
| **LI-RADS ancillary features** | | |
| ***Favoring malignancy, not HCC in particular*** | | |
| Diffusion restriction (present vs. absent) | Signal intensity of the tumor higher than the liver on diffusion-weighted images not caused only by T2 shine-through. (1,2) | High b value (e.g.,≥800) DWI |
| Fat in mass, more than liver (present vs. absent) | Excess fat within a mass, in whole or in part, relative to the adjacent liver. (1,2) | In-/opposed phase; fat-suppressed images compared to non-fat-suppressed images with similar or identical weighting |
| Iron sparing in solid mass (present vs. absent) | Paucity of iron in solid mass relative to iron-overloaded liver OR in inner nodule relative to siderotic outer nodule. (1,2) | In-/opposed phase; T2-weighted image (T2WI) |
| Mild-moderate T2WI hyperintensity (present vs. absent) | Signal intensity of the tumor on T2WI higher than the liver, similar to or lower than a non-iron-overloaded spleen, and lower than simple fluid. (1,2) | T2WI |
| Corona enhancement (present vs. absent) | Peritumoral enhancement in the late arterial phase or early portal venous phase. The enhancement is contiguous with and surrounds all or part of the tumor. (1,2) | Late arterial or early portal venous phase |
| ***Favoring malignancy, HCC in particular*** | | |
| Nonenhancing capsule (present vs. absent) | Subtype of capsule that does not show enhancement on any image. (1,2) | Multiple sequences |
| Nodule-in-nodule architecture (present vs. absent) | Presence of smaller inner nodule within and having different imaging features than larger outer nodule. | Multiple sequences |
| Mosaic architecture (present vs. absent) | Presence of any combination of internal nodules, compartments, or septations, within a solid or mostly solid mass. (1,2) | Multiple sequences |
| Blood products in mass (present vs. absent) | Intralesional or perilesional hemorrhage in the absence of biopsy, trauma, or intervention. (1,2) | Multiple sequences |
| ***LR-M features*** | | |
| Rim APHE (present vs. absent) | Spatially defined subtype of arterial phase hyperenhancement in which arterial phase enhancement is most pronounced in tumor periphery. (1,2) | Late arterial phase |
| Delayed central enhancement (present vs. absent) | Central area of progressive postarterial phase enhancement. (1,2) | Postarterial phases |
| Targetoid restriction (present vs. absent) | Concentric pattern on diffusion-weighted imaging characterized by restricted diffusion in tumor periphery with less restricted diffusion in tumor center. (1,2) | DWI or ADC map |
| Marked diffusion restriction (present vs. absent) | Signal intensity of the tumor similar to or higher than non-iron-overloaded spleen on diffusion-weighted images not caused only by T2 shine-through. (1,2) | High b value (e.g.,≥800) DWI |
| Infiltrative appearance (present vs. absent) | Presence of a non-circumscribed tumor margin (indistinct transition) thought to represent a permeative growth pattern. (1,2) | Multiple sequences |
| Necrosis or severe ischemia (present vs. absent) | Presence of nonenhancing area in a solid mass, not attributable to a cystic component, prior treatment, or intralesional hemorrhage. (1,2) | Post-contrast phases and T2WI |
| **Other tumor-related prognostic features** | | |
| T2WI peritumoral hyperintensity (present vs. absent) | Presence of irregular, wedge-shaped, or flame-like area adjacent to the tumor on T2WI of which the signal intensity is mildly or moderately higher than liver and similar to or less than a non-iron-overloaded spleen. (3,4) | T2WI |
| Portal venous phase (PVP) peritumoral hypoenhancement (present vs. absent) | Presence of irregular, wedge-shaped, or flame-like hypoenhancing area adjacent to the tumor in the portal venous phase. (3,4) | Portal venous phase |
| Low apparent diffusion coefficient (ADC) value (present vs. absent) | Apparent diffusion coefficient value of the tumor in whole or in part lower than the liver. | ADC map |
| APHE proportion (≤50% vs. >50%) | The proportion of tumor volume demonstrating arterial phase hyperenhancement. (5) | Late arterial phase |
| Intratumoral artery (present vs. absent) | Presence of discrete arteries within the tumor on arterial phase images. (3,6,7) | Early or late arterial phase |
| Complete capsule (present vs. absent) | Presence of non-disrupted “capsule” in all imaging planes. (3,8) | Multiple sequences |
| Non-smooth tumor margin (present vs. absent) | The tumor margin is irregular and/or has areas of bulging, nodular projection, or infiltration into adjacent tissues at the tumor periphery in any imaging plane. (3,7,9) | Multiple sequences |
| Involvement of liver capsule (present vs. absent) | Unequivocal tumor involvement of liver capsule. | Multiple sequences |
| Bilobar involvement (present vs. absent) | Unequivocal tumor involvement of more than one liver lobes. (10) | Multiple sequences |
| The VICT2 trait (present vs. absent) | Presence of portal venous phase peritumoral hypoenhancement; or presence of corona enhancement, T2-weighted peritumoral hyperintensity coupled with absence of complete capsule. (4) | Multiple sequences |
| Tumor growth subtype |  | Multiple sequences |
| Simple nodular type | A round expanding nodule with a distinct margin in all imaging planes. (13,14) |  |
| Non-simple nodular type | An expanding tumor with areas of bulging or nodular extranodular projection, a cluster of small and confluent nodules, or the presence of an indistinct margin throughout the entire tumor. (13,14) |  |
| **Imaging features associated with the severity of underlying liver diseases and portal hypertension** | | |
| Ascites (present vs. absent) | Presence of free fluid in abdomen or pelvis. (11) | T2WI |
| Cirrhosis (present vs. absent) | Unequivocal morphological alterations of the liver, including surface nodularity, small liver volume, expansion of space between liver and anterior abdominal wall and perihilar, gallbladder fossa and ligamentum teres spaces, hypertrophy of caudate and/or left lateral section, atrophy of anterior right section and/or left medial section, anterolateral flattening, notching of posterior medial right lobe and parenchymal nodules, with or without manifestations of portal hypertension (portal-systemic collaterals, splenomegaly and/or ascites). (1,2) | Multiple sequences |
| Diffuse iron overload (present vs. absent) | Diffuse signal intensity drop of the liver parenchyma on in-phase images compared with the opposed-phase images. (1,2) | In-/opposed phase; T2WI |
| Liver steatosis (present vs. absent) | Diffuse signal intensity drop of the liver parenchyma on opposed-phase images compared with the in-phase images. (1,2) | In-/opposed phase; fat-suppressed images compared to non-fat-suppressed images with similar or identical weighting |
| Splenomegaly (present vs. absent) | Diameter of the spleen>12cm on axial image. (11,12,13) | Multiple sequences |

**References:**

1. CT/MRI Liver Imaging Reporting and Data System version 2018. American College of Radiology Web site. [https://www.acr.org/Clinical-Resources/Reporting-andData- Systems/LI-RADS/CTMRI-LI-RADS-v2018. Accessed 1 September 2021](https://www.acr.org/Clinical-Resources/Reporting-andData-%20Systems/LI-RADS/CTMRI-LI-RADS-v2018.%20Accessed%201%20September%202021).
2. LI-RADS Lexicon (terms and definitions). American College of Radiology Web site. <https://www.acr.org/-/media/ACR/Files/RADS/LI-RADS/LIRADS-Lexicon-Table.pdf>. Accessed 1 September 2021.
3. Jiang H, Wei J, Fu F, et al. Predicting microvascular invasion in hepatocellular carcinoma: a dual-institution study on gadoxetate disodium-enhanced MRI. Liver Int 2022;42(5):1158–1172.
4. Jiang H, Wei H, Yang T, et al. VICT2 Trait: Prognostic Alternative to Peritumoral Hepatobiliary Phase Hypointensity in HCC. Radiology 2023 Apr;307(2):e221835.
5. Rhee H, Cho ES, Nahm JH, et al. Gadoxetic acid-enhanced MRI of macrotrabecular-massive hepatocellular carcinoma and its prognostic implications. J Hepatol 2021;74(1):109–121.
6. Segal E, Sirlin CB, Ooi C, et al. Decoding global gene expression programs in liver cancer by noninvasive imaging. Nat Biotechnol 2007;25(6):675–680.
7. Renzulli M, Brocchi S, Cucchetti A, et al. Can Current Preoperative Imaging Be Used to Detect Microvascular Invasion of Hepatocellular Carcinoma? Radiology 2016;279(2):432–442.
8. Lei Z, Li J, Wu D, et al. Nomogram for Preoperative Estimation of Microvascular Invasion Risk in Hepatitis B Virus-Related Hepatocellular Carcinoma Within the Milan Criteria. JAMA Surg 2016;151(4):356–363.
9. An C, Kim DW, Park YN, et al. Single hepatocellular carcinoma: preoperative MR imaging to predict early recurrence after curative resection. Radiology 2015;276(2):433–443.
10. Wei H, Fu F, Jiang H, et al. Development and validation of the OSASH score to predict overall survival of hepatocellular carcinoma after surgical resection: a dual-institutional study. Eur Radiol 2023;33(11):7631–7645.
11. Venkatesh SK, Yin M, Takahashi N, et al. Non-invasive detection of liver fibrosis: MR imaging features vs. MR elastography. Abdom Radiol (NY) 2015;40(4):766–775.
12. Rustogi R, Horowitz J, Harmath C, et al. Accuracy of MR elastography and anatomic MR imaging features in the diagnosis of severe hepatic fibrosis and cirrhosis. J Magn Reson Imaging 2012;35(6):1356–1364.
13. Marasco G, Colecchia A, Colli A, et al. Role of liver and spleen stiffness in predicting the recurrence of hepatocellular carcinoma after resection. J Hepatol 2019;70(3):440–448.

**Table S3. Inter- and intra-rater agreement in nonperipheral washout pattern**

| **Group / Assessment** | **Outcome type** | **Readers** | **κ (95% CI)** | **Agreement** |
| --- | --- | --- | --- | --- |
| ***Inter-rater agreement*** | | | | |
| Synthesized nonperipheral washout patterns (Round 1 & 2) | no / early / late washout | All three readers | 0.70 (0.66 – 0.73) | Substantial |
| Observed nonperipheral washout patterns (Round 3) | no / early / late washout | All three readers | 0.74 (0.70 – 0.77) | Substantial |
| Early washout (Round 1, pre-contrast and PVP) | present / absent | All three readers | 0.67 (0.62 – 0.72) | Substantial |
| Late washout (Round 2, pre-contrast and DP) | present / absent | All three readers | 0.70 (0.66 – 0.75) | Substantial |
| Early washout (Round 3, precontrast, PVP, and DP) | present / absent | All three readers | 0.70 (0.66 – 0.75) | Substantial |
| Late washout (Round 3, precontrast, PVP, and DP) | present / absent | All three readers | 0.74 (0.70 – 0.79) | Substantial |
| ***Intra-rater agreement*** | | | | |
| Nonperipheral washout pattern (Synthesized vs Observed) | no / early / late washout | R1 | 0.84 (0.81 – 0.87) | Excellent |
| Nonperipheral washout pattern (Synthesized vs Observed) | no / early / late washout | R2 | 0.82 (0.78 – 0.85) | Excellent |
| Nonperipheral washout pattern (Synthesized vs Observed) | no / early / late washout | R3 | 0.81 (0.78 – 0.84) | Excellent |
| Early washout (Round 1 vs Round 3) | present / absent | R1 | 0.85 (0.82 – 0.88) | Excellent |
| Late washout (Round 2 vs Round 3) | present / absent | R1 | 0.94 (0.92 – 0.96) | Excellent |
| Early washout (Round 1 vs Round 3) | present / absent | R2 | 0.88 (0.86 – 0.91) | Excellent |
| Late washout (Round 2 vs Round 3) | present / absent | R2 | 0.84 (0.81 – 0.87) | Excellent |
| Early washout (Round 1 vs Round 3) | present / absent | R3 | 0.86 (0.84 – 0.89) | Excellent |
| Late washout (Round 2 vs Round 3) | present / absent | R3 | 0.84 (0.81 – 0.87) | Excellent |

Note.— All κ values are based on 611 lesions. Synthesized washout pattern was analyzed based on integration of phase-specific evaluations from Rounds 1 and 2; Observed washout pattern was analyzed based on direct temporal classification from Round 3. κ interpreted per Landis–Koch criteria. Agreement was considered poor (ICC or κ< 0), slight (ICC or κ, 0-0.20), fair (ICC or κ, 0.20-0.40), moderate (ICC or κ, 0.40-0.60), substantial (ICC or κ, 0.60-0.80), or excellent (ICC or κ> 0.80).

Abbreviations: ICC = intraclass correlation coefficient, PVP = portal venous phase, DP = delayed phase.

**Table S4. Inter-rater agreement for all evaluated imaging features**

| **Features** | **κ or ICC**^*^ | **Agreement** |
| --- | --- | --- |
| LI RADS category (LR-4/LR-5/LR-M)* | 0.52 (0.47, 0.56) | moderate |
| **LI-RADS major features** | | |
| Nonrim APHE | 0.59 (0.53, 0.65) | moderate |
| Nonperipheral washout | 0.58 (0.52, 0.64) | moderate |
| Enhancing capsule | 0.31 (0.25, 0.37) | fair |
| Tumor size (cm) | 0.98 (0.97, 0.98) | excellent |
| **LI-RADS ancillary features** | | |
| ***Favoring malignancy, not HCC in particular*** | | |
| Corona enhancement | 0.40 (0.35, 0.46) | moderate |
| Fat in mass more than liver | 0.49 (0.43, 0.55) | moderate |
| Mild to moderate T2WI hyperintensity | 0.38 (0.32, 0.44) | fair |
| Diffusion restriction | 0.27 (0.21, 0.32) | fair |
| Iron sparing in solid mass | 0.51 (0.45, 0.57) | moderate |
| ***Favoring malignancy, HCC in particular*** | | |
| Nonenhancing capsule | 0.32 (0.26, 0.38) | fair |
| Nodule-in-nodule architecture | 0.45 (0.39, 0.51) | moderate |
| Mosaic architecture | 0.59 (0.53, 0.64) | moderate |
| Blood products in mass | 0.79 (0.73, 0.85) | substantial |
| ***LR-M features*** | | |
| Rim APHE | 0.65 (0.59, 0.71) | substantial |
| Delayed central enhancement | 0.42 (0.37, 0.48) | moderate |
| Targetoid restriction | 0.39 (0.33, 0.44) | fair |
| Marked diffusion restriction | 0.45 (0.39, 0.50) | moderate |
| Infiltrative appearance | 0.25 (0.19, 0.31) | fair |
| Necrosis or severe ischemia | 0.74 (0.68, 0.79) | substantial |
| **Other tumor-related prognostic features** | | |
| T2WI peritumoral hyperintensity | 0.57 (0.51, 0.63) | moderate |
| PVP peritumoral hypoenhancement | 0.58 (0.52, 0.64) | moderate |
| Low ADC value | 0.79 (0.48, 0.83) | substantial |
| APHE proportion (≤50% vs. >50%) | 0.54 (0.49, 0.60) | moderate |
| Intratumoral artery | 0.53 (0.48, 0.59) | moderate |
| Complete capsule | 0.41 (0.35, 0.47) | moderate |
| Non-smooth tumor margin | 0.51 (0.45, 0.57) | moderate |
| The VICT2 trait^†^ | 0.56 (0.51, 0.62) | moderate |
| Tumor growth subtype | 0.47 (0.42, 0.53) | moderate |
| Bilobar involvement | 0.59 (0.53, 0.65) | moderate |
| Involvement of liver capsule | 0.70 (0.64, 0.75) | substantial |
| **Imaging features associated with the severity of underlying liver diseases and portal hypertension** | | |
| Ascites | 0.42 (0.36, 0.47) | moderate |
| Cirrhosis | 0.44 (0.38, 0.49) | moderate |
| Liver steatosis | 0.68 (0.62, 0.74) | substantial |
| Diffuse iron overload | 0.54 (0.48, 0.60) | moderate |
| Splenomegaly | 0.55 (0.49, 0.60) | moderate |

Note.— Data in parentheses are 95% confidence intervals.

Inter-rater agreement was assessed with the intraclass correlation coefficient (ICC) for tumor size, with the weighted κ value for LI-RADS categories, and with the Fleiss' κ value for categorical imaging features. Agreement was considered poor (κ or ICC < 0.0), slight (κ or ICC: 0.0-0.2), fair (κ or ICC: 0.2-0.4), moderate (κ or ICC: 0.4-0.6), substantial (κ or ICC: 0.6-0.8), or excellent (κ or ICC > 0.8).

^*^LI-RADS categories were reported only for patients with the LI-RADS high-risk status (n=567).

^†^The VICT2 trait is defined as the presence of portal venous phase peritumoral hypoenhancement; or the presence of both corona enhancement and T2-weighted peritumoral hyperintensity coupled with the absence of a complete capsule

Abbreviations: ICC=intraclass correlation coefficient, LI-RADS = Liver Imaging Reporting and Data System, T2WI = T2-weighted imaging, APHE = arterial phase hyperenhancement, PVP = portal venous phase, ADC = apparent diffusion coefficient.

**Table S5. Differences in imaging features according to nonperipheral washout pattern**

| **Features** | **No Washout (n = 149)** | **Late Washout  (n = 95)** | | **Early Washout (n = 367)** | P value **overall** | P value^*^  (No vs Late) | P value^*^  (No vs Early) | P value^*^  (Late vs Early) |
| --- | --- | --- | --- | --- | --- | --- | --- | --- |
| **LI-RADS major features** | | | | | | | | |
| Nonrim APHE | 144 (96.6) | 93 (97.9) | 335 (91.3) | | .**014** | >.99 | .036 | **.030** |
| Nonperipheral washout^†^ | 0 (0.0) | 95 (100.0) | 367 (100.0) | | **<.001** | **<.001** | **<.001** | .74 |
| Enhancing capsule | 125 (83.9) | 91 (95.8) | 362 (98.6) | | **<.001** | **<.001** | **<.001** | .41 |
| Tumor size (cm) | 2.4 (1.8, 3.0) | 2.8 (2.2, 4.0) | 3.8 (2.5, 6.0) | | **<.001** | **.013** | **<.001** | **<.001** |
| **LI-RADS ancillary features** | | | | | | | | |
| ***Favoring malignancy, not HCC in particular*** | | | | | | | | |
| Corona enhancement | 43 (28.9) | 21 (22.1) | 149 (40.6) | | <.001 | .42 | .017 | **.001** |
| Fat in mass more than liver | 48 (32.2) | 34 (35.8) | 173 (47.1) | | .**003** | .87 | .003 | .07 |
| Mild to moderate T2WI hyperintensity | 146 (98.0) | 94 (99.0) | 359 (97.8) | | .92 | - | - | - |
| Diffusion restriction | 148 (99.3) | 92 (96.8) | 366 (99.7) | | .**033** | - | - | - |
| Iron sparing in solid mass | 14 (9.4) | 10 (10.5) | 23 (6.3) | | .26 | - | - | - |
| ***Favoring malignancy, HCC in particular*** | | | | | | | | |
| Nonenhancing capsule^†^ | 5 (3.4) | 0 (0.0) | 0 (0.0) | | .002 | .007 | <.001 | >.99 |
| Nodule in nodule architecture | 18 (12.1) | 24 (25.3) | 147 (40.1) | | <.001 | .045 | <.001 | **.008** |
| Mosaic architecture | 11 (7.4) | 8 (8.4) | 117 (31.9) | | <.001 | >.99 | <.001 | **<.001** |
| Blood products in mass | 16 (10.7) | 11 (11.6) | 113 (30.8) | | <.001 | >.99 | <.001 | **<.001** |
| ***LR-M features*** | | | | | | | | |
| Rim APHE^†^ | 3 (2.0) | 2 (2.1) | 28 (7.6) | | .**011** | >.99 | .016 | .05 |
| Delayed central enhancement^†^ | 5 (3.4) | 1 (1.1) | 9 (2.5) | | .53 | - | - | - |
| Targetoid restriction^†^ | 1 (0.7) | 1 (1.1) | 9 (2.5) | | .47 | - | - | - |
| Marked diffusion restriction | 36 (24.2) | 15 (15.8) | 99 (27.0) | | .08 | - | - | - |
| Infiltrative appearance^†^ | 0 (0.0) | 0 (0.0) | 9 (2.5) | | .06 | - | - | - |
| Necrosis or severe ischemia | 11 (7.4) | 21 (22.1) | 158 (43.1) | | **<.001** | **.023** | **<.001** | **<.001** |
| ***LI RADS category^‡^*** | | | | | | | | |
| LR-4 | 48 (34.5) | 3 (3.4) | 3 (0.9) | | **<.001** | **<.001** | **<.001** | .06 |
| LR-5 | 85 (61.2) | 82 (94.3) | 306 (89.7) | |  |  |  |  |
| LR-M | 6 (4.3) | 2 (2.3) | 32 (9.4) | |  |  |  |  |
| **Other tumor-related prognostic features** | | | | | | | | |
| T2WI peritumoral hyperintensity | 16 (10.7) | 12 (12.6) | 93 (25.3) | | **<.001** | >.99 | **<.001** | **.008** |
| PVP peritumoral hypoenhancement | 6 (4.0) | 6 (6.3) | 98 (26.7) | | **<.001** | .98 | **<.001** | **<.001** |
| Low ADC value§ | 51 (38.9) | 28 (33.7) | 174 (53.2) | | **<.001** | .69 | **.009** | **.002** |
| APHE proportion>50% | 142 (95.3) | 91 (95.8) | 259 (70.6) | | **<.001** | >.99 | **<.001** | **<.001** |
| Intratumoral artery | 8 (5.4) | 9 (9.5) | 115 (31.3) | | **<.001** | .67 | **<.001** | **<.001** |
| Complete capsule | 74 (49.7) | 47 (49.5) | 89 (24.3) | | **<.001** | >.99 | **<.001** | **<.001** |
| Non-smooth tumor margin | 83 (55.7) | 57 (6.0) | 297 (80.9) | | **<.001** | .70 | **<.001** | **<.001** |
| The VICT2 trait^¶^ | 24 (16.1) | 17 (17.9) | 145 (39.5) | | **<.001** | >.99 | **<.001** | **<.001** |
| Tumor growth subtype |  |  |  | |  |  |  |  |
| Simple nodular type | 117 (78.5) | 62 (65.3) | 152 (41.4) | | **<.001** | .07 | **<.001** | **<.001** |
| Non-simple nodule type | 32 (21.5) | 33 (34.7) | 215 (58.6) | |  |  |  |  |
| Bilobar involvement^†^ | 5 (3.4) | 2 (2.1) | 16 (4.4) | | .56 | - | - | - |
| Involvement of liver capsule | 88 (59.1) | 59 (62.1) | 249 (67.9) | | .14 | - | - | - |
| **Imaging features associated with the severity of underlying liver diseases and portal hypertension** | | | | | | | | |
| Ascites | 10 (6.7) | 3 (3.2) | 27 (7.4) | | .34 |  |  |  |
| Cirrhosis | 92 (61.7) | 58 (61.1) | 222 (60.5) | | .97 |  |  |  |
| Liver steatosis | 36 (24.2) | 9 (9.5) | 68 (18.5) | | **.016** | **.006** | .20 | .06 |
| Diffuse iron overload | 23 (15.4) | 13 (13.7) | 36 (9.8) | | .16 |  |  |  |
| Splenomegaly | 60 (40.3) | 40 (42.1) | 153 (41.7) | | .34 |  |  |  |

Note.— Data are numbers of patients, with percentages in parentheses, or medians, with IQRs in parentheses. Unless stated otherwise, comparisons were made using the χ² test for categorical variables and the Kruskal-Wallis test for continuous variables.

^*^For characteristics with significant differences across all three groups (*P* All < .05), post hoc pairwise comparisons were performed with Bonferroni correction for categorical variables and Dunn's test for continuous and ordinal variables.

^†^Comparisons were made using the Fisher exact test.

^‡^Comparisons were made in patients with LI-RADS high-risk status (No washout, n=139; Late washout, n=87; Early washout, n=341).

§Data available for 541 patients (No washout, n=131; Late washout, n=83; Early washout, n=327).

^¶^The VICT2 trait is defined as the presence of portal venous phase peritumoral hypoenhancement; or the presence of both corona enhancement and T2-weighted peritumoral hyperintensity coupled with the absence of a complete capsule.

Abbreviations: LI-RADS = Liver Imaging Reporting and Data System, T2WI = T2-weighted imaging, APHE = arterial phase hyperenhancement, PVP = portal venous phase, ADC = apparent diffusion coefficient.

**Table S6. Univariable Cox regression analyses for early and 5-year recurrence-free survival among all patients**

| **Variable** | **Early Recurrence-Free Survival** | |  | **5-year Recurrence-Free Survival** | |
| --- | --- | --- | --- | --- | --- |
|  | **Univariable HR** | ***P value*** |  | **Univariable HR** | ***P value*** |
| **Washout classification**^*^ | | |  |  |  |
| Early washout (present vs absent) | 2.29 (1.59, 3.28) | **<.001** |  | 1.69 (1.30, 2.22) | **<.001** |
| Conventional washout (present vs absent) | 2.19 (1.39, 3.42) | **<.001** |  | 1.82 (1.31, 2.54) | **<.001** |
| **Imaging** | | |  |  |  |
| ***LI-RADS major features*** | | |  |  |  |
| Nonrim APHE (present vs absent) | 0.79 (0.44, 1.42) | .43 |  | 0.64 (0.41, 0.98) | **.042** |
| Enhancing capsule (present vs absent) | 1.57 (0.69, 3.54) | .28 |  | 1.09 (0.62, 1.90) | .77 |
| Tumor size (cm) | 1.14 (1.09, 1.19) | **<.001** |  | 1.11 (1.06, 1.15) | **<.001** |
| Tumor size (>2 cm vs ≤2 cm) | 1.80 (1.13, 2.88) | **.014** |  | 1.40 (0.99, 1.97) | .05 |
| Tumor size (>3 cm vs ≤3 cm) | 1.99 (1.43, 2.76) | **<.001** |  | 1.57 (1.22, 2.02) | **<.001** |
| Tumor size (>5 cm vs ≤5 cm) | 1.85 (1.33, 2.57) | **<.001** |  | 1.57 (1.20, 2.06) | **.001** |
| ***LI-RADS ancillary features*** | | |  |  |  |
| *Favoring malignancy, not HCC in particular* | | |  |  |  |
| Corona enhancement (present vs absent) | 1.17 (0.85, 1.61) | .34 |  | 0.96 (0.74, 1.25) | .74 |
| Fat in mass more than liver (present vs absent) | 0.90 (0.64, 1.26) | .53 |  | 0.89 (0.69, 1.15) | .39 |
| Mild to moderate T2WI hyperintensity (present vs absent) | 3.62 (0.51, 25.83) | .20 |  | 0.60 (0.30, 1.21) | .16 |
| Diffusion restriction (present vs absent) | 1.42 (0.20, 10.13) | .73 |  | 1.02 (0.25, 4.10) | .98 |
| Iron sparing in solid mass (present vs absent) | 1.27 (0.74, 2.21) | .39 |  | 1.03 (0.64, 1.64) | .91 |
| *Favoring malignancy, HCC in particular* | | |  |  |  |
| Nonenhancing capsule (present vs absent) | 3.06 (0.97, 9.59) | .06 |  | 2.16 (0.69, 6.76) | .18 |
| Nodule in nodule architecture (present vs absent) | 0.95 (0.68, 1.34) | .77 |  | 0.88 (0.67, 1.16) | .38 |
| Mosaic architecture (present vs absent) | 1.62 (1.15, 2.28) | **.006** |  | 1.45 (1.10, 1.92) | **.009** |
| Blood products in mass (present vs absent) | 1.69 (1.19, 2.40) | **.003** |  | 1.71 (1.30, 2.24) | **<.001** |
| ***LR-M features*** | | |  |  |  |
| At least one LR-M feature (present vs absent) | 1.63 (1.02, 2.61) | **.041** |  | 1.58 (1.07, 2.33) | **.020** |
| Rim APHE (present vs absent) | 1.60 (0.89, 2.88) | .12 |  | 1.68 (1.06, 2.69) | **.029** |
| Delayed central enhancement (present vs absent) | 0.72 (0.23, 2.26) | .58 |  | 1.10 (0.52, 2.33) | .81 |
| Targetoid restriction (present vs absent) | 1.53 (0.57, 4.13) | .40 |  | 1.45 (0.64, 3.25) | .37 |
| Marked diffusion restriction (present vs absent) | 1.28 (0.91, 1.82) | .16 |  | 1.05 (0.79, 1.41) | .72 |
| Infiltrative appearance (present vs absent) | 1.85 (0.68, 5.00) | .23 |  | 1.75 (0.72, 4.25) | .21 |
| Necrosis or severe ischemia (present vs absent) | 1.53 (1.10, 2.14) | **.010** |  | 1.63 (1.27, 2.11) | **<.001** |
| ***Other tumor-related prognostic features*** | | |  |  |  |
| T2WI peritumoral hyperintensity (present vs absent) | 1.62 (1.14, 2.29) | **.007** |  | 1.30 (0.97, 1.76) | .08 |
| PVP peritumoral hypoenhancement (present vs absent) | 1.81 (1.27, 2.57) | **<.001** |  | 1.43 (1.05, 1.93) | **.021** |
| Low ADC value (present vs absent) | 1.19 (0.86, 1.65) | .29 |  | 1.05 (0.81, 1.36) | .70 |
| APHE proportion (>50% vs ≤50%) | 0.65 (0.46, 0.94) | **.021** |  | 0.66 (0.50, 0.88) | **.005** |
| Intratumoral artery (present vs absent) | 2.16 (1.55, 3.00) | **<.001** |  | 1.70 (1.29, 2.24) | **<.001** |
| Complete capsule (absent vs present) | 1.67 (1.11, 2.51) | **.010** |  | 1.51 (1.15, 1.99) | **.003** |
| Non-smooth tumor margin (present vs absent) | 1.93 (1.29, 2.88) | **.001** |  | 1.63 (1.20, 2.20) | **.002** |
| The VICT2 trait (present vs absent) | 1.50 (1.09, 2.06) | **.013** |  | 1.13 (0.87, 1.48) | .35 |
| Tumor growth subtype (non-simple nodular vs simple nodular) | 2.02 (1.47, 2.78) | **<.001** |  | 1.59 (1.24, 2.04) | **<.001** |
| Bilobar involvement (present vs absent) | 1.44 (0.67 ~ 3.06) | .35 |  | 0.85 (0.42, 1.71) | .64 |
| Involvement of liver capsule (present vs absent) | 1.40 (1.00, 1.98) | .05 |  | 1.44 (1.09, 1.89) | **.01** |
| **Clinical** | | |  |  |  |
| Age (y) | 0.98 (0.97, 1.00) | **.008** |  | 0.99 (0.98, 1.00) | **.017** |
| Sex (M vs F) | 0.97 (0.63, 1.50) | .90 |  | 1.01 (0.71, 1.43) | .95 |
| ALBI grade (grade 2 vs grade 1) | 1.10 (0.83, 1.46) | .49 |  | 1.05 (0.78, 1.40) | .74 |
| BCLC stage (0 vs A) | 1.32 (0.96, 1.83) | .09 |  | 1.31 (0.94, 1.82) | .11 |
| α-fetoprotein (ng/mL) | 1.00 (1.00, 1.00) | .10 |  | 1.00 (1.00, 1.00) | .26 |
| α-fetoprotein (ng/mL)^†^ | 1.18 (1.11, 1.25) | **<.001** |  | 1.12 (1.07, 1.18) | **<.001** |
| α-fetoprotein (>100 ng/mL vs ≤100 ng/mL) | 1.96 (1.43, 2.67) | **<.001** |  | 1.58 (1.23, 2.03) | **<.001** |
| α-fetoprotein (>400 ng/mL vs ≤400 ng/mL) | 1.98 (1.43, 2.74) | **<.001** |  | 1.73 (1.32, 2.26) | **<.001** |
| **Surgical** | | |  |  |  |
| Open surgery (yes vs no) | 1.71 (1.17, 2.50) | **.005** |  | 1.48 (1.11, 1.97) | **.007** |
| Major hepatectomy (yes vs no) | 1.11 (0.69, 1.80) | .66 |  | 0.92 (0.62, 1.39) | .71 |
| **Histopathologic** |  |  |  |  |  |
| Microvascular invasion (present vs absent) | 3.17 (2.31, 4.35) | **<.001** |  | 2.63 (2.05, 3.38) | **<.001** |
| Edmondson-Steiner grade (III/IV vs I/II) | 1.42 (1.03, 1.95) | **.030** |  | 1.18 (0.91, 1.53) | .20 |

Note.— Data in parentheses are 95% CIs.

^*^Early washout was defined as nonperipheral washout observed in the portal venous phase (regardless of the findings in the delayed phase); conventional washout was defined as nonperipheral washout observed in either the portal venous phase or the delayed phase; while late washout was defined as nonperipheral washout observed in the delayed phase and not in the portal venous phase.

^†^Data were computed after logarithmic transformation.

Abbreviations: LI-RADS = Liver Imaging Reporting and Data System, T2WI = T2-weighted imaging, APHE = arterial phase hyperenhancement, PVP = portal venous phase, ADC = apparent diffusion coefficient.

**Table S7. Cox regression analyses for early recurrence-free survival among patients with available satellite nodule data**

| **Variable** | **Early Recurrence-Free Survival** | | | |
| --- | --- | --- | --- | --- |
|  | **Univariable HR** | ***P value*** | **Multivariable HR** | ***P value*** |
| **Washout classification**^*^ | | |  |  |
| Early washout (present vs absent) | 3.02 (1.68, 5.40) | **<.001** | 1.94 (1.19, 3.16) | **.008** |
| Conventional washout (present vs absent) | 2.68 (1.33, 5.38) | **.006** | ... | ... |
| **Imaging** | | |  |  |
| ***LI-RADS major features*** | | |  |  |
| Nonrim APHE (present vs absent) | 0.57 (0.26, 1.23) | .15 | ... | ... |
| Enhancing capsule (present vs absent) | 1.38 (0.43, 4.38) | .59 | ... | ... |
| Tumor size (cm) | 1.12 (1.03, 1.21) | **.005** |  |  |
| Tumor size (>2 cm vs ≤2 cm) | 1.65 (0.89, 3.07) | .11 | ... | ... |
| Tumor size (>3 cm vs ≤3 cm) | 1.69 (1.05, 2.73) | **.03** | ... | ... |
| Tumor size (>5 cm vs ≤5 cm) | 1.28 (0.77, 2.15) | .34 | ... | ... |
| ***LI-RADS ancillary features*** | | |  |  |
| ***Favoring malignancy, not HCC in particular*** | | |  |  |
| Corona enhancement (present vs absent) | 0.92 (0.56, 1.49) | .73 | ... | ... |
| Fat in mass more than liver (present vs absent) | 0.88 (0.55, 1.41) | .61 | ... | ... |
| Mild to moderate T2WI hyperintensity (present vs absent) | 9.30 ×10^06 (0.00, Inf) | .99 | ... | ... |
| Diffusion restriction (present vs absent) | 0.65 (0.09, 4.65) | .66 | ... | ... |
| Iron sparing in solid mass (present vs absent) | 0.90 (0.36, 2.22) | .81 | ... | ... |
| ***Favoring malignancy, HCC in particular*** | | |  |  |
| Nonenhancing capsule (present vs absent) | 2.72 (0.38, 19.57) | .32 | ... | ... |
| Nodule in nodule architecture (present vs absent) | 0.56 (0.32, 0.97) | **.04** | 0.62 (0.39, 0.98) | **.04** |
| Mosaic architecture (present vs absent) | 1.14 (0.67, 1.93) | .64 | ... | ... |
| Blood products in mass (present vs absent) | 1.54 (0.94, 2.52) | .09 | ... | ... |
| ***LR-M features*** | | |  |  |
| Rim APHE (present vs absent) | 2.20 (1.01, 4.80) | .05 | ... | ... |
| Delayed central enhancement (present vs absent) | 0.47 (0.06, 3.36) | .45 | ... | ... |
| Targetoid restriction (present vs absent) | 0.82 (0.11, 5.88) | .84 | ... | ... |
| Marked diffusion restriction (present vs absent) | 1.60 (0.98, 2.63) | .06 | ... | ... |
| Infiltrative appearance (present vs absent) | 2.54 (0.62, 10.38) | .19 | ... | ... |
| Necrosis or severe ischemia (present vs absent) | 1.39 (0.87, 2.23) | .17 | ... | ... |
| ***Other tumor-related prognostic features*** | | |  |  |
| T2WI peritumoral hyperintensity (present vs absent) | 1.68 (0.99, 2.82) | .05 | ... | ... |
| PVP peritumoral hypoenhancement (present vs absent) | 1.51 (0.89, 2.57) | .13 | ... | ... |
| Low ADC value (present vs absent) | 1.21 (0.77, 1.91) | .41 | ... | ... |
| APHE proportion (>50% vs ≤50%) | 0.87 (0.49, 1.54) | .64 | ... | ... |
| Intratumoral artery (present vs absent) | 1.50 (0.91, 2.46) | .11 | ... | ... |
| Complete capsule (present vs absent) | 0.61 (0.37, 1.02) | .06 | ... | ... |
| Non-smooth tumor margin (present vs absent) | 1.66 (0.94, 2.93) | .08 | ... | ... |
| The VICT2 trait (present vs absent) | 1.34 (0.84, 2.14) | .22 | ... | ... |
| Tumor growth subtype (non-simple nodular vs simple nodular) | 1.98 (1.24, 3.14) | **.004** | ... | ... |
| Bilobar involvement (present vs absent) | 1.43 (0.45, 4.54) | .55 | ... | ... |
| Involvement of liver capsule (present vs absent) | 1.28 (0.78, 2.11) | .33 | ... | ... |
| **Clinical** | | |  |  |
| Age (y) | 0.98 (0.96, 1.00) | .07 | ... | ... |
| Sex (M vs F) | 0.70 (0.40, 1.22) | .21 | ... | ... |
| ALBI grade (grade 2 vs grade 1) | 0.81 (0.45, 1.48) | .50 | ... | ... |
| α-fetoprotein (ng/mL) | 1.00 (1.00, 1.00) | .93 | ... | ... |
| α-fetoprotein (ng/mL)^†^ | 1.24 (1.15, 1.35) | **<.001** | 1.20 (1.11, 1.30) | **<.001** |
| α-fetoprotein (>100 ng/mL vs ≤100 ng/mL) | 3.04 (1.92, 4.80) | **<.001** | ... | ... |
| α-fetoprotein (>400 ng/mL vs ≤400 ng/mL) | 3.09 (1.94, 4.93) | **<.001** | ... | ... |
| **Surgical** | | |  |  |
| Open surgery (yes vs no) | 1.25 (0.77, 2.05) | .37 | ... | ... |
| Major hepatectomy (yes vs no) | 1.09 (0.53, 2.28) | .81 | ... | ... |
| **Histopathologic** |  |  |  |  |
| Microvascular invasion (present vs absent) | 2.66 (1.69, 4.20) | **<.001** | 1.90 (1.23, 2.92) | **.004** |
| Edmondson-Steiner grade (III/IV vs I/II) | 1.71 (1.07, 2.71) | **.02** | ... | ... |
| Satellite nodules (present vs absent) | 2.14 (1.07, 4.31) | **.03** | ... | ... |
| Note.—Data in parentheses are 95% CIs. After accounting for high collinearity , 8 features (early washout, tumor size, nodule in nodule architecture, tumor growth subtype, α-fetoprotein†, microvascular invasion, Edmondson–Steiner grade, satellite nodules) were included in the multivariable Cox regression model to evaluate early recurrence-free survival.  *Early washout was defined as nonperipheral washout observed in the portal venous phase (regardless of the findings in the delayed phase), while conventional washout was defined as nonperipheral washout observed in either the portal venous phase or the delayed phase. †Data were computed after logarithmic transformation. | | | | |

**Table S8. Cox regression analyses for 5-year recurrence-free survival among patients with available satellite nodule data**

| **Variable** | **5-year Recurrence-Free Survival** | | | |
| --- | --- | --- | --- | --- |
|  | **Univariable HR** | ***P value*** | **Multivariable HR** | ***P value*** |
| **Washout classification**^*^ | | |  |  |
| Early washout (present vs absent) | 1.9 (1.29, 2.81) | **.001** | 1.62 (1.12, 2.34) | **.01** |
| Conventional washout (present vs absent) | 2.2 (1.35, 3.58) | **.002** | ... | ... |
| **Imaging** | | |  |  |
| ***LI-RADS major features*** | | |  |  |
| Nonrim APHE (present vs absent) | 0.63 (0.34, 1.17) | .14 |  |  |
| Enhancing capsule (present vs absent) | 0.82 (0.40, 1.67) | .58 | ... | ... |
| Tumor size (cm) | 1.06 (0.99, 1.13) | .08 | ... | ... |
| Tumor size (>2 cm vs ≤2 cm) | 1.35 (0.87, 2.11) | .19 | ... | ... |
| Tumor size (>3 cm vs ≤3 cm) | 1.29 (0.91, 1.84) | .16 | ... | ... |
| Tumor size (>5 cm vs ≤5 cm) | 1.05 (0.69, 1.59) | .82 | ... | ... |
| ***LI-RADS ancillary features*** | | |  |  |
| ***Favoring malignancy, not HCC in particular*** | | |  |  |
| Corona enhancement (present vs absent) |  |  |  |  |
| Fat in mass more than liver (present vs absent) | 0.93 (0.65, 1.32) | .68 | ... | ... |
| Mild to moderate T2WI hyperintensity (present vs absent) | 0.79 (0.29, 2.14) | .64 | ... | ... |
| Diffusion restriction (present vs absent) | 1.05 (0.15, 7.48) | .97 | ... | ... |
| Iron sparing in solid mass (present vs absent) | 0.85 (0.43, 1.67) | .63 | ... | ... |
| ***Favoring malignancy, HCC in particular*** | | |  |  |
| Nonenhancing capsule (present vs absent) | 1.76 (0.25, 12.61) | .57 | ... | ... |
| Nodule in nodule architecture (present vs absent) | 0.56 (0.37, 0.85) | **.007** | 0.53 (0.36, 0.78) | **.001** |
| Mosaic architecture (present vs absent) | 1.06 (0.70, 1.61) | .77 | ... | ... |
| Blood products in mass (present vs absent) | 1.48 (1.01, 2.16) | .05 | ... | ... |
| ***LR-M features*** | | |  |  |
| Rim APHE (present vs absent) | 2.18 (1.17, 4.05) | **.01** | ... | ... |
| Delayed central enhancement (present vs absent) | 0.82 (0.26, 2.56) | .73 | ... | ... |
| Targetoid restriction (present vs absent) | 0.85 (0.21, 3.44) | .82 | ... | ... |
| Marked diffusion restriction (present vs absent) | 1.35 (0.92, 2.00) | .13 | ... | ... |
| Infiltrative appearance (present vs absent) | 1.33 (0.33, 5.37) | .69 | ... | ... |
| Necrosis or severe ischemia (present vs absent) | 1.10 (0.75, 1.61) | .62 | ... | ... |
| ***Other tumor-related prognostic features*** | | |  |  |
| T2WI peritumoral hyperintensity (present vs absent) | 1.38 (0.90, 2.11) | .14 | ... | ... |
| PVP peritumoral hypoenhancement (present vs absent) | 1.23 (0.79, 1.90) | .36 | ... | ... |
| Low ADC value (present vs absent) | 1.09 (0.77, 1.55) | .62 | ... | ... |
| APHE proportion (>50% vs ≤50%) | 0.91 (0.59, 1.41) | .68 | ... | ... |
| Intratumoral artery (present vs absent) |  |  |  |  |
| Complete capsule (present vs absent) | 0.83 (0.57, 1.20) | .32 | ... | ... |
| Non-smooth tumor margin (present vs absent) | 1.47 (0.97, 2.24) | .07 | ... | ... |
| The VICT2 trait (present vs absent) | 1.05 (0.72, 1.54) | .78 | ... | ... |
| Tumor growth subtype (non-simple nodular vs simple nodular) | 1.35 (0.95, 1.92) | .09 | ... | ... |
| Bilobar involvement (present vs absent) | 0.6 (0.19, 1.89) | .38 | ... | ... |
| Involvement of liver capsule (present vs absent) | 1.48 (1.00, 2.18) | .05 | ... | ... |
| **Clinical** | | |  |  |
| Age (y) | 0.99 (0.98, 1.01) | .24 | ... | ... |
| Sex (M vs F) |  |  |  |  |
| ALBI grade (grade 2 vs grade 1) | 0.97 (0.66, 1.43) | .88 |  |  |
| α-fetoprotein (ng/mL) | 1.00 (1.00, 1.00) | .56 | ... | ... |
| α-fetoprotein (ng/mL)^†^ | 1.16 (1.09, 1.23) | **<.001** | 1.13 (1.06, 1.20) | **<.001** |
| α-fetoprotein (>100 ng/mL vs ≤100 ng/mL) | 2.17 (1.52, 3.08) | **<.001** | ... | ... |
| α-fetoprotein (>400 ng/mL vs ≤400 ng/mL) | 2.32 (1.58, 3.39) | **<.001** | ... | ... |
| **Surgical** | | |  |  |
| Open surgery (yes vs no) | 1.21 (0.84, 1.76) | .31 | ... | ... |
| Major hepatectomy (yes vs no) | 0.99 (0.55, 1.79) | .97 | ... | ... |
| **Histopathologic** |  |  |  |  |
| Microvascular invasion (present vs absent) | 2.17 (1.53, 3.09) | **<.001** | 1.62 (1.14, 2.31) | **.007** |
| Edmondson-Steiner grade (III/IV vs I/II) | 1.29 (0.90, 1.86) | .17 | ... | ... |
| Satellite nodules (present vs absent) | 2.79 (1.65, 4.73) | **<.001** | 2.26 (1.33, 3.84) | **.003** |
| Note.—Data in parentheses are 95% CIs. After accounting for high collinearity, 6 features (early washout, nodule in nodule architecture, rim APHE, α-fetoprotein†, microvascular invasion, satellite nodules) were included in the multivariable Cox regression model to evaluate 5-year recurrence-free survival.  *Early washout was defined as nonperipheral washout observed in the portal venous phase (regardless of the findings in the delayed phase), while conventional washout was defined as nonperipheral washout observed in either the portal venous phase or the delayed phase. †Data were computed after logarithmic transformation. | | | | |

**Table S9. Cox regression analyses for early recurrence-free survival in LI-RADS high-risk population**

| **Variable** | **Early Recurrence-Free Survival** | | | |
| --- | --- | --- | --- | --- |
|  | **Univariable HR** | ***P value*** | **Multivariable HR** | ***P value*** |
| **Washout classification**^*^ | | |  |  |
| Early washout (present vs absent) | 2.32 (1.61, 3.37) | **<.001** | 1.91 (1.31, 2.78) | **<.001** |
| Conventional washout (present vs absent) | 2.10 (1.33, 3.29) | **.001** | ... | ... |
| **Imaging** | | |  |  |
| ***LI-RADS major features*** | | |  |  |
| Nonrim APHE (present vs absent) | 0.8 (0.44, 1.44) | .45 | ... | ... |
| Enhancing capsule (present vs absent) | 1.64 (0.73, 3.72) | .23 | ... | ... |
| Tumor size (cm) | 1.15 (1.09, 1.20) | **<.001** | ... | ... |
| Tumor size (>2 cm vs ≤2 cm) | 1.86 (1.17, 2.98) | **.009** | ... | ... |
| Tumor size (>3 cm vs ≤3 cm) | 2.24 (1.60, 3.14) | **<.001** | ... | ... |
| Tumor size (>5 cm vs ≤5 cm) | 2.01 (1.43, 2.81) | **<.001** | ... | ... |
| ***LI-RADS ancillary features*** | | |  |  |
| ***Favoring malignancy, not HCC in particular*** | | |  |  |
| Corona enhancement (present vs absent) | 1.23 (0.88, 1.70) | .22 | ... | ... |
| Fat in mass more than liver (present vs absent) | 0.92 (0.67, 1.28) | .63 | ... | ... |
| Mild to moderate T2WI hyperintensity (present vs absent) | 3.44 (0.48, 24.56) | .22 | ... | ... |
| Diffusion restriction (present vs absent) | 1.14 (0.16, 8.18) | .89 | ... | ... |
| Iron sparing in solid mass (present vs absent) | 1.38 (0.80, 2.40) | .25 | ... | ... |
| ***Favoring malignancy, HCC in particular*** | | |  |  |
| Nonenhancing capsule (present vs absent) | 2.92 (0.93, 9.18) | .07 | ... | ... |
| Nodule in nodule architecture (present vs absent) | 0.98 (0.69, 1.40) | .93 | ... | ... |
| Mosaic architecture (present vs absent) | 1.70 (1.20, 2.40) | **.003** | ... | ... |
| Blood products in mass (present vs absent) | 2.00 (1.43, 2.80) | **<.001** | ... | ... |
| ***LR-M features*** | | |  |  |
| Rim APHE (present vs absent) | 1.60 (0.89, 2.88) | .12 | ... | ... |
| Delayed central enhancement (present vs absent) | 0.82 (0.26, 2.57) | .73 | ... | ... |
| Targetoid restriction (present vs absent) | 1.67 (0.62, 4.51) | .31 | ... | ... |
| Marked diffusion restriction (present vs absent) | 1.3 (0.91, 1.86) | .14 | ... | ... |
| Infiltrative appearance (present vs absent) | 2.05 (0.76, 5.53) | .16 | ... | ... |
| Necrosis or severe ischemia (present vs absent) | 2.03 (1.48, 2.80) | **<.001** | ... | ... |
| ***Other tumor-related prognostic features*** | | |  |  |
| T2WI peritumoral hyperintensity (present vs absent) | 1.59 (1.11, 2.28) | **.01** | ... | ... |
| PVP peritumoral hypoenhancement (present vs absent) | 1.89 (1.32, 2.72) | **<.001** | ... | ... |
| Low ADC value (present vs absent) | 1.29 (0.92, 1.80) | .13 | ... | ... |
| APHE proportion (>50% vs ≤50%) | 0.66 (0.46, 0.95) | **.03** | ... | ... |
| Intratumoral artery (present vs absent) | 2.20 (1.57, 3.09) | **<.001** | ... | ... |
| Complete capsule (absent vs present) | 1.92 (1.32, 2.80) | **<.001** | ... | ... |
| Non-smooth tumor margin (present vs absent) | 1.97 (1.31, 2.97) | **.001** | ... | ... |
| The VICT2 trait (present vs absent) | 1.54 (1.11, 2.14) | **.01** | ... | ... |
| Tumor growth subtype (non-simple nodular vs simple nodular) | 2.01 (1.45, 2.77) | **<.001** | ... | ... |
| Bilobar involvement (present vs absent) | 1.47 (0.65, 3.32) | .36 | ... | ... |
| Involvement of liver capsule (present vs absent) | 1.01 (0.73, 1.39) | .96 | ... | ... |
| **Clinical** | | |  |  |
| Age (y) | 0.99 (0.97, 1.00) | .05 | ... | ... |
| Sex (M vs F) | 0.96 (0.62,1.50) | .86 | ... | ... |
| ALBI grade (grade 2 vs grade 1) | 0.92 (0.63, 1.36) | .69 | ... | ... |
| α-fetoprotein (ng/mL) | 1.00 (1.00, 1.00) | **.04** | ... | ... |
| α-fetoprotein (ng/mL)^†^ | 1.18 (1.11, 1.26) | **<.001** | 1.12 (1.05,1.19) | **<.001** |
| α-fetoprotein (>100 ng/mL vs ≤100 ng/mL) | 1.96 (1.43, 2.69) | **<.001** | ... | ... |
| α-fetoprotein (>400 ng/mL vs ≤400 ng/mL) | 1.99 (1.43, 2.77) | **<.001** | ... | ... |
| **Surgical** | | |  |  |
| Open surgery (yes vs no) | 1.56 (1.06, 2.28) | **.02** | ... | ... |
| Major hepatectomy (yes vs no) | 1.13 (0.69, 1.84) | .64 | ... | ... |
| **Histopathologic** |  |  |  |  |
| Microvascular invasion (present vs absent) | 3.04 (2.20, 4.20) | **<.001** | 2.40 (1.71, 3.36) | **<.001** |
| Edmondson-Steiner grade (III/IV vs I/II) | 1.33 (0.97, 1.84) | .08 | ... | ... |
| Note.—Data in parentheses are 95% CIs. After accounting for high collinearity (i.e., known clinical collinearity, such as continuous and categorical forms of laboratory indices, Spearman’s rho > 0.6, or a variance inflation factor > 5), 13 features (early washout, tumor size > 3cm, blood products in mass, necrosis or severe ischemia, PVP peritumoral hypoenhancement, T2WI peritumoral hyperintensity, APHE proportion, complete capsule, intratumoral artery, tumor growth subtype, α-fetoprotein†, open surgery, microvascular invasion) were included in the multivariable Cox regression model to evaluate early recurrence-free survival.  *Early washout was defined as nonperipheral washout observed in the portal venous phase (regardless of the findings in the delayed phase), while conventional washout was defined as nonperipheral washout observed in either the portal venous phase or the delayed phase. †Data were computed after logarithmic transformation. | | | | |

**Table S10. Cox regression analyses for 5-year recurrence-free survival in LI-RADS high-risk population**

| **Variable** | **5-year Recurrence-Free Survival** | | | |
| --- | --- | --- | --- | --- |
|  | **Univariable HR** | ***P value*** | **Multivariable HR** | ***P value*** |
| **Washout classification**^*^ |  |  |  |  |
| Early washout (present vs absent) | 1.71 (1.30, 2.25) | **<.001** | 1.49 (1.13, 1.97) | **.005** |
| Conventional washout (present vs absent) | 1.78 (1.27, 2.49) | **<.001** | ... | ... |
| **Imaging** |  |  |  |  |
| ***LI-RADS major features*** |  |  |  |  |
| Nonrim APHE (present vs absent) | 0.65 (0.41, 1.01) | .06 | ... | ... |
| Enhancing capsule (present vs absent) | 1.09 (0.62, 1.92) | .75 | ... | ... |
| Tumor size (cm) | 1.11 (1.06, 1.16) | **<.001** | ... | ... |
| Tumor size (>2 cm vs ≤2 cm) | 1.43 (1.01, 2.01) | **.04** | ... | ... |
| Tumor size (>3 cm vs ≤3 cm) | 1.70 (1.31, 2.20) | **<.001** | ... | ... |
| Tumor size (>5 cm vs ≤5 cm) | 1.63 (1.23, 2.16) | **<.001** | ... | ... |
| ***LI-RADS ancillary features*** |  |  |  |  |
| ***Favoring malignancy, not HCC in particular*** |  |  |  |  |
| Corona enhancement (present vs absent) | 1.00 (0.75, 1.33) | >.99 | ... | ... |
| Fat in mass more than liver (present vs absent) | 0.91 (0.70, 1.18) | .48 | ... | ... |
| Mild to moderate T2WI hyperintensity (present vs absent) | 0.55 (0.27, 1.12) | .10 | ... | ... |
| Diffusion restriction (present vs absent) | 0.53 (0.13, 2.15) | .38 | ... | ... |
| Iron sparing in solid mass (present vs absent) | 1.13 (0.70, 1.80) | .62 | ... | ... |
| ***Favoring malignancy, HCC in particular*** |  |  |  |  |
| Nonenhancing capsule (present vs absent) | 2.09 (0.67, 6.52) | .21 | ... | ... |
| Nodule in nodule architecture (present vs absent) | 0.91 (0.68, 1.21) | .52 | ... | ... |
| Mosaic architecture (present vs absent) | 1.47 (1.10, 1.96) | **.009** | ... | ... |
| Blood products in mass (present vs absent) | 1.71 (1.29, 2.26) | **<.001** | ... | ... |
| ***LR-M features*** |  |  |  |  |
| Rim APHE (present vs absent) | 1.6 (0.99, 2.58) | .06 | ... | ... |
| Delayed central enhancement (present vs absent) | 1.06 (0.47, 2.39) | .89 | ... | ... |
| Targetoid restriction (present vs absent) | 1.6 (0.71, 3.60) | .26 | ... | ... |
| Marked diffusion restriction (present vs absent) | 1.08 (0.79, 1.48) | .61 | ... | ... |
| Infiltrative appearance (present vs absent) | 1.69 (0.70, 4.11) | .24 | ... | ... |
| Necrosis or severe ischemia (present vs absent) | 1.63 (1.26, 2.13) | **<.001** | ... | ... |
| ***Other tumor-related prognostic features*** |  |  |  |  |
| T2WI peritumoral hyperintensity (present vs absent) | 1.26 (0.93, 1.72) | .14 | ... | ... |
| PVP peritumoral hypoenhancement (present vs absent) | 1.47 (1.07, 2.01) | **.02** | ... | ... |
| Low ADC value (present vs absent) | 1.09 (0.84, 1.42) | .52 | ... | ... |
| APHE proportion (>50% vs ≤50%) | 0.74 (0.54, 1.00) | .05 | ... | ... |
| Intratumoral artery (present vs absent) | 1.68 (1.26, 2.24) | **<.001** | ... | ... |
| Complete capsule (absent vs present) | 1.47 (1.11, 1.95) | **.007** | ... | ... |
| Non-smooth tumor margin (present vs absent) | 1.59 (1.17, 2.16) | **.003** | ... | ... |
| The VICT2 trait (present vs absent) | 1.14 (0.87, 1.51) | .34 | ... | ... |
| Tumor growth subtype (non-simple nodular vs simple nodular) | 1.56 (1.21, 2.01) | **<.001** | ... | ... |
| Bilobar involvement (present vs absent) | 0.89 (0.42, 1.88) | .76 | ... | ... |
| Involvement of liver capsule (present vs absent) | 1.53 (1.16, 2.03) | **.003** | ... | ... |
| **Clinical** |  |  |  |  |
| Age (y) | 0.99 (0.98, 1.00) | .08 | ... | ... |
| Sex (M vs F) | 1.07 (0.74, 1.56) | .71 | ... | ... |
| ALBI grade (grade 2 vs grade 1) | 1.08 (0.80, 1.46) | .61 | ... | ... |
| α-fetoprotein (ng/mL) | 1.00 (1.00, 1.00) | .11 | ... | ... |
| α-fetoprotein (ng/mL)^†^ | 1.13 (1.07, 1.19) | **<.001** | 1.09 (1.03, 1.14) | .001 |
| α-fetoprotein (>100 ng/mL vs ≤100 ng/mL) | 1.58 (1.23, 2.04) | **<.001** | ... | ... |
| α-fetoprotein (>400 ng/mL vs ≤400 ng/mL) | 1.74 (1.32, 2.28) | **<.001** | ... | ... |
| **Surgical** |  |  |  |  |
| Open surgery (yes vs no) | 1.46 (1.09, 1.97) | **.01** | ... | ... |
| Major hepatectomy (yes vs no) | 0.98 (0.65, 1.49) | .94 | ... | ... |
| **Histopathologic** |  |  |  |  |
| Microvascular invasion (present vs absent) | 2.49 (1.93, 3.22) | **<.001** | 2.16 (1.66, 2.82) | <.001 |
| Edmondson-Steiner grade (III/IV vs I/II) | 1.13 (0.86, 1.47) | .38 | ... | ... |
| Note.—Data in parentheses are 95% CIs. After accounting for high collinearity (i.e., known clinical collinearity, such as continuous and categorical forms of laboratory indices, Spearman’s rho > 0.6, or a variance inflation factor > 5), 11 features (Early washout, Tumor size, Mosaic architecture, Blood products in mass, PVP peritumoral hypoenhancement, Non-smooth tumor margin, Tumor growth subtype, Involvement of liver capsule, α-fetoprotein†, open surgery, microvascular invasion) were included in the multivariable Cox regression model to evaluate 5-year recurrence-free survival.  *Early washout was defined as nonperipheral washout observed in the portal venous phase (regardless of the findings in the delayed phase), while conventional washout was defined as nonperipheral washout observed in either the portal venous phase or the delayed phase. †Data were computed after logarithmic transformation. | | | | |

**Table S11. Impact of early washout on 5-year recurrence-free survival according to different subgroups**

| **Subgroup** | **No. of events (%)*** | **Univariable analysis** | | |  | **Multivariable analysis adjusted for the other predictive variables** | | | |
| --- | --- | --- | --- | --- | --- | --- | --- | --- | --- |
|  |  | **HR (95% CI)** | ***P* value** | ***P* for interaction** |  | **HR (95% CI)** | ***P* value** | ***P* for interaction** | **Variables adjusted for** |
| **AFP** |  |  |  | >.99 |  |  |  | .37 |  |
| α-fetoprotein ≤ 400 ng/mL | 114 (42.7) | 1.66 (1.21-2.28) | **.002** |  |  | 1.60 (1.16-2.20) | **.004** |  | MVI |
| α-fetoprotein > 400 ng/mL | 57 (57.0) | 1.59 (0.96-2.63) | .07 |  |  | 1.18 (0.69-2.01) | .54 |  | MVI |
| **Tumor size** |  |  |  | .15 |  |  |  | .05 |  |
| Tumor size ≤ 2 cm | 21 (50.0) | 2.56 (1.36-4.81) | **.004** |  |  | 2.59 (1.36-4.92) | **.004** |  | MVI, LnAFP |
| Tumor size > 2 cm | 150 (46.2) | 1.48 (1.10-2.00) | **.01** |  |  | 1.27 (0.94-1.72) | .12 |  | MVI, LnAFP |
| **Microvascular invasion** |  |  |  | **.03** |  |  |  | .07 |  |
| absent | 67 (31.6) | 1.15 (0.80-1.65) | .44 |  |  | 1.19 (0.83-1.70) | .36 |  | LnAFP |
| present | 104 (67.1) | 2.11 (1.36-3.26) | **.001** |  |  | 2.03 (1.31-3.16) | **.002** |  | LnAFP |
| **Edmondson-Steiner grade** |  |  |  | .17 |  |  |  | .23 |  |
| I/II | 99 (44.8) | 1.50 (1.09-2.05) | **.01** |  |  | 1.35 (0.98-1.86) | .07 |  | MVI, LnAFP |
| III/IV | 72 (49.3) | 2.22 (1.29-3.82) | **.004** |  |  | 1.85 (1.07-3.22) | **.03** |  | MVI, LnAFP |
| **Cirrhosis** |  |  |  | .35 |  |  |  | .67 |  |
| absent | 51 (35.2) | 2.09 (1.22-3.57) | **.007** |  |  | 1.62 (0.93-2.84) | .09 |  | MVI, LnAFP |
| present | 120 (54.1) | 1.59 (1.17-2.17) | **.003** |  |  | 1.49 (1.09-2.03) | **.01** |  | MVI, LnAFP |
| **Liver steatosis** |  |  |  | .30 |  |  |  | .30 |  |
| absent | 143 (47.8) | 1.60 (1.20-2.13) | **.001** |  |  | 1.39 (1.04-1.87) | **.03** |  | MVI, LnAFP |
| present | 28 (41.2) | 2.38 (1.12-5.04) | **.02** |  |  | 2.01 (0.94-4.29) | .07 |  | MVI, LnAFP |
| **Enhancing capsule** |  |  |  | .52 |  |  |  | .60 |  |
| present | 169 (46.7) | 1.76 (1.32-2.33) | **<.001** |  |  | 1.51 (1.14-2.02) | **.005** |  | MVI, LnAFP |
| **MRI field strength** |  |  |  | .49 |  |  |  | .42 |  |
| 1.5 T | 103 (54.2) | 1.56 (1.11-2.19) | **.01** |  |  | 1.33 (0.94-1.87) | .11 |  | MVI, LnAFP |
| 3.0 T | 68 (38.4) | 1.90 (1.23-2.96) | **.004** |  |  | 1.69 (1.08-2.65) | **.02** |  | MVI, LnAFP |
| **Resection extent** |  |  |  | .51 |  |  |  | .21 |  |
| minor resection | 150 (47.9) | 1.77 (1.34-2.34) | **<.001** |  |  | 1.59 (1.20-2.11) | **.001** |  | MVI, LnAFP |
| **Resection type** |  |  |  | .29 |  |  |  |  |  |
| laparoscopic surgery | 37 (35.2) | 1.33 (0.80-2.21) | .27 |  |  | 1.24 (0.74-2.07) | .41 |  | MVI, LnAFP |
| Open surgery | 134 (51.1) | 1.82 (1.32-2.50) | **<.001** |  |  | 1.54 (1.11-2.13) | **.009** |  | MVI, LnAFP |
| **Resection margin** |  |  |  | **.03** |  |  |  | .05 |  |
| Resection margin < 1 cm | 27 (37.5) | 3.81 (1.66-8.76) | **.002** |  |  | 3.27 (1.41-7.58) | **.006** |  | MVI, LnAFP |
| Resection margin ≥ 1 cm | 58 (43.3) | 1.31 (0.84-2.05) | .23 |  |  | 1.31 (0.84-2.05) | .23 |  | MVI, LnAFP |
| Note.—Data in parentheses are 95% CIs.   * No. of events (%) in patients with early washout. | | | | | | | | | |

**Table S12. Uni- and multivariable Fine-Gray competing risks analyses for time to early recurrence**

| **Variable** |  | **Time to Early Recurrence** | | | | | |
| --- | --- | --- | --- | --- | --- | --- | --- |
|  | **Univariable Fine-Gray Analysis** | | |  | **Multivariable Fine-Gray Analysis** | | |
|  | **β Value** | **sHR** | ***P value*** |  | **β Value** | **sHR** | ***P value*** |
| **Washout classification**^*^ | | | |  |  |  |  |
| Early washout (present vs absent) | 0.84 | 2.32 (1.59, 3.37) | **<.001** |  | 0.65 | 1.92 (1.31, 2.81) | **<.001** |
| Conventional washout (present vs absent) | 0.75 | 2.12 (1.33, 3.38) | **.002** |  | ... | ... | ... |
| **Imaging** | | | |  |  |  |  |
| ***LI-RADS major features*** | | | |  |  |  |  |
| Nonrim APHE (present vs absent) | -0.31 | 0.73 (0.40, 1.34) | .31 |  | ... | ... | ... |
| Enhancing capsule (present vs absent) | 0.56 | 1.76 (0.71, 4.34) | .22 |  | ... | ... | ... |
| Tumor size (cm) | 0.12 | 1.13 (1.07, 1.19) | **<.001** |  | ... | ... | ... |
| Tumor size (>2 cm vs ≤2 cm) | 0.50 | 1.65 (1.03, 2.64) | **.04** |  | ... | ... | ... |
| Tumor size (>3 cm vs ≤3 cm) | 0.60 | 1.83 (1.31, 2.56) | **<.001** |  | ... | ... | ... |
| Tumor size (>5 cm vs ≤5 cm) | 0.59 | 1.80 (1.28, 2.54) | **<.001** |  | ... | ... | ... |
| ***LI-RADS ancillary features*** | | | |  |  |  |  |
| ***Favoring malignancy, not HCC in particular*** | | | |  |  |  |  |
| Corona enhancement (present vs absent) | 0.08 | 1.08 (0.77, 1.51) | .66 |  | ... | ... | ... |
| Fat in mass more than liver (present vs absent) | -0.02 | 0.98 (0.71, 1.36) | .91 |  | ... | ... | ... |
| Mild to moderate T2WI hyperintensity (present vs absent) | 1.217 | 3.35 (0.45, 25.21) | .24 |  | ... | ... | ... |
| Diffusion restriction (present vs absent) | 0.27 | 1.31 (0.18, 9.44) | .79 |  | ... | ... | ... |
| Iron sparing in solid mass (present vs absent) | 0.13 | 1.14 (0.63, 2.04) | .67 |  | ... | ... | ... |
| ***Favoring malignancy, HCC in particular*** | | | |  |  |  |  |
| Nonenhancing capsule (present vs absent) | 1.19 | 3.27 (1.04, 10.34) | **.04** |  | ... | ... | ... |
| Nodule in nodule architecture (present vs absent) | -0.15 | 0.86 (0.60, 1.23) | .40 |  | ... | ... | ... |
| Mosaic architecture (present vs absent) | 0.44 | 1.56 (1.09, 2.23) | **.02** |  | ... | ... | ... |
| Blood products in mass (present vs absent) | 0.60 | 1.83 (1.29, 2.58) | <.001 |  | ... | ... | ... |
| ***LR-M features*** | | | |  |  |  |  |
| Rim APHE (present vs absent) | 0.55 | 1.73 (0.94, 3.18) | .08 |  | ... | ... | ... |
| Delayed central enhancement (present vs absent) | -0.70 | 0.50 (0.12, 2.04) | .33 |  | ... | ... | ... |
| Targetoid restriction (present vs absent) | 0.50 | 1.65 (0.66, 4.11) | .29 |  | ... | ... | ... |
| Marked diffusion restriction (present vs absent) | 0.28 | 1.32 (0.92, 1.89) | .13 |  | ... | ... | ... |
| Infiltrative appearance (present vs absent) | 0.83 | 2.29 (0.82, 6.38) | .11 |  | ... | ... | ... |
| Necrosis or severe ischemia (present vs absent) | 0.60 | 1.83 (1.32, 2.53) | **<.001** |  | ... | ... | ... |
| ***Other tumor-related prognostic features*** | | | |  |  |  |  |
| T2WI peritumoral hyperintensity (present vs absent) | 0.38 | 1.47 (1.02, 2.12) | **.04** |  | ... | ... | ... |
| PVP peritumoral hypo-enhancement (present vs absent) | 0.58 | 1.78 (1.23, 2.58) | **.002** |  | ... | ... | ... |
| Low ADC value (present vs absent) | 0.21 | 1.24 (0.88, 1.74) | .21 |  | ... | ... | ... |
| APHE proportion (>50% vs ≤50%) | -0.35 | 0.70 (0.48, 1.03) | .07 |  | ... | ... | ... |
| Intratumoral artery (present vs absent) | 0.67 | 1.95 (1.37, 2.76) | **<.001** |  | ... | ... | ... |
| Complete capsule (present vs absent) | -0.60 | 0.55 (0.38, 0.80) | **.002** |  | ... | ... | ... |
| Non-smooth tumor margin (present vs absent) | 0.61 | 1.85 (1.23, 2.77) | **.003** |  | ... | ... | ... |
| The VICT2 trait (present vs absent) | 0.36 | 1.44 (1.03, 2.00) | **.03** |  | ... | ... | ... |
| Tumor growth subtype (non-simple nodular vs simple nodular) | 0.64 | 1.89 (1.37, 2.62) | **<.001** |  | ... | ... | ... |
| Bilobar involvement (present vs absent) | 0.23 | 1.26 (0.58, 2.75) | .56 |  | ... | ... | ... |
| Involvement of liver capsule (present vs absent) | 0.34 | 1.41 (0.99, 2.00) | .06 |  | ... | ... | ... |
| **Clinical** | | | |  |  |  |  |
| Age (y) | -0.02 | 0.98 (0.97, 1.00) | **.02** |  | ... | ... | ... |
| Sex (M vs F) | -0.12 | 0.89 (0.58, 1.38) | .60 |  | ... | ... | ... |
| ALBI grade (grade 2 vs grade 1) | -0.20 | 0.82 (0.55, 1.23) | .34 |  | ... | ... | ... |
| α-fetoprotein (ng/mL) | 0 | 1.00 (1.00, 1.00) | .17 |  | ... | ... | ... |
| α-fetoprotein (ng/mL)^†^ | 0.16 | 1.17 (1.10, 1.24) | **<.001** |  | 0.11 | 1.11 (1.04, 1.19) | **.001** |
| α-fetoprotein(>100 ng/mL vs ≤100 ng/mL) | 0.66 | 1.94 (1.41, 2.68) | **<.001** |  | ... | ... | ... |
| α-fetoprotein(>400 ng/mL vs ≤400 ng/mL) | 0.66 | 1.93 (1.37, 2.72) | **<.001** |  | ... | ... | ... |
| **Surgical** | | | |  |  |  |  |
| Open surgery (yes vs no) | 0.44 | 1.55 (1.06, 2.27) | **.02** |  | ... | ... | ... |
| Major hepatectomy (yes vs no) | 0.19 | 1.21 (0.75, 1.95) | .44 |  | ... | ... | ... |
| **Histopathologic** |  |  |  |  |  |  |  |
| Microvascular invasion (present vs absent) | 1.04 | 2.83 (2.05, 3.91) | **<.001** |  | 0.81 | 2.24 (1.59, 3.15) | **<.001** |
| Edmondson-Steiner grade (III/IV vs I/II) | 0.35 | 1.41 (1.02, 1.96) | **.04** |  | ... | ... | ... |
| Note.—Data in parentheses are 95% CIs. After accounting for multicollinearity, 15 variables (early washout, tumor size >3, nonenhancing capsule, mosaic architecture, necrosis or severe ischemia, T2WI peritumoral hyperintensity, PVP peritumoral hypoenhancement, intratumoral artery, non-smooth tumor margin, tumor growth subtype, age, α-fetoprotein†, open surgery, microvascular invasion, Edmondson–Steiner grade) underwent multivariable analysis. sHR = subdistribution hazard ratio. †Data were computed after logarithmic transformation. | | | | | | | |

**Table S13. Uni- and multivariable Fine-Gray competing risks analyses for time to 5-year recurrence**

| **Variable** |  | **Time to 5-year Recurrence** | | | | | |
| --- | --- | --- | --- | --- | --- | --- | --- |
|  | **Univariable Fine-Gray Analysis** | | |  | **Multivariable Fine-Gray Analysis** | | |
|  | **β Value** | **sHR** | ***P value*** |  | **β Value** | **sHR** | ***P value*** |
| **Washout classification**^*^ | | | |  |  |  |  |
| Early washout (present vs absent) | 0.50 | 1.65 (1.25, 2.18) | **<.001** |  | 0.39 | 1.48 (1.12, 1.96) | **.007** |
| Conventional washout (present vs absent) | 0.49 | 1.64 (1.17, 2.30) | **.004** |  | ... | ... | ... |
| **Imaging** | | | |  |  |  |  |
| ***LI-RADS major features*** | | | |  |  |  |  |
| Nonrim APHE (present vs absent) | -0.42 | 0.66 (0.41, 1.05) | .08 |  | ... | ... | ... |
| Enhancing capsule (present vs absent) | 0.25 | 1.29 (0.69, 2.40) | .43 |  | ... | ... | ... |
| Tumor size (cm) | 0.08 | 1.09 (1.03, 1.14) | **.002** |  | ... | ... | ... |
| Tumor size (>2 cm vs ≤2 cm) | 0.24 | 1.27 (0.90, 1.79) | .18 |  | ... | ... | ... |
| Tumor size (>3 cm vs ≤3 cm) | 0.32 | 1.37 (1.05, 1.79) | **.02** |  | ... | ... | ... |
| Tumor size (>5 cm vs ≤5 cm) | 0.39 | 1.47 (1.09, 1.98) | **.01** |  | ... | ... | ... |
| ***LI-RADS ancillary features*** | | | |  |  |  |  |
| ***Favoring malignancy, not HCC in particular*** | | | |  |  |  |  |
| Corona enhancement (present vs absent) | -0.16 | 0.85 (0.64, 1.13) | .26 |  | ... | ... | ... |
| Fat in mass more than liver (present vs absent) | -0.11 | 0.90 (0.69, 1.18) | .44 |  | ... | ... | ... |
| Mild to moderate T2WI hyperintensity (present vs absent) | -0.31 | 0.74 (0.36, 1.50) | .40 |  | ... | ... | ... |
| Diffusion restriction (present vs absent) |  |  |  |  |  |  |  |
| Iron sparing in solid mass (present vs absent) | -0.06 | 0.94 (0.56, 1.58) | .82 |  | ... | ... | ... |
| ***Favoring malignancy, HCC in particular*** | | | |  |  |  |  |
| Nonenhancing capsule (present vs absent) | 0.90 | 2.45 (0.66, 9.13) | .18 |  | ... | ... | ... |
| Nodule in nodule architecture (present vs absent) | -0.31 | 0.74 (0.54, 1.00) | .05 |  | ... | ... | ... |
| Mosaic architecture (present vs absent) | 0.32 | 1.37 (1.01, 1.86) | **.04** |  | ... | ... | ... |
| Blood products in mass (present vs absent) | 0.49 | 1.63 (1.22, 2.18) | **.001** |  | ... | ... | ... |
| ***LR-M features*** | | | |  |  |  |  |
| Rim APHE (present vs absent) | 0.46 | 1.58 (0.93, 2.68) | .09 |  | ... | ... | ... |
| Delayed central enhancement (present vs absent) | -0.4 | 0.66 (0.25, 1.73) | .40 |  | ... | ... | ... |
| Targetoid restriction (present vs absent) | 0.04 | 1.04 (0.38, 2.89) | .93 |  | ... | ... | ... |
| Marked diffusion restriction (present vs absent) | 0.15 | 1.16 (0.85, 1.57) | .34 |  | ... | ... | ... |
| Infiltrative appearance (present vs absent) | 0.41 | 1.50 (0.49, 4.59) | .48 |  | ... | ... | ... |
| Necrosis or severe ischemia (present vs absent) | 0.38 | 1.46 (1.11, 1.92) | **.007** |  | ... | ... | ... |
| ***Other tumor-related prognostic features*** | | | |  |  |  |  |
| T2WI peritumoral hyperintensity (present vs absent) | 0.16 | 1.17 (0.84, 1.63) | .34 |  | ... | ... | ... |
| PVP peritumoral hypoenhancement (present vs absent) | 0.34 | 1.40 (1.00, 1.96) | .05 |  | ... | ... | ... |
| Low ADC value (present vs absent) | 0.10 | 1.10 (0.84, 1.45) | .49 |  | ... | ... | ... |
| APHE proportion (>50% vs ≤50%) | -0.24 | 0.79 (0.57, 1.09) | .14 |  | ... | ... | ... |
| Intratumoral artery (present vs absent) | 0.39 | 1.47 (1.08, 2.01) | **.01** |  | ... | ... | ... |
| Complete capsule (present vs absent) | -0.34 | 0.71 (0.54, 0.95) | **.02** |  | ... | ... | ... |
| Non-smooth tumor margin (present vs absent) | 0.39 | 1.48 (1.09, 2.01) | **.01** |  | ... | ... | ... |
| The VICT2 trait (present vs absent) | 0.10 | 1.11 (0.83, 1.48) | .49 |  | ... | ... | ... |
| Tumor growth subtype (non-simple nodular vs simple nodular) | 0.34 | 1.40 (1.08, 1.82) | **.01** |  | ... | ... | ... |
| Bilobar involvement (present vs absent) | -0.21 | 0.81 (0.38, 1.74) | .59 |  | ... | ... | ... |
| Involvement of liver capsule (present vs absent) | 0.36 | 1.43 (1.07, 1.91) | **.02** |  | ... | ... | ... |
| **Clinical** | | | |  |  |  |  |
| Age (y) | -0.01 | 0.99 (0.98, 1.00) | **.02** |  | ... | ... | ... |
| Sex (M vs F) |  |  |  |  |  |  |  |
| ALBI grade (grade 2 vs grade 1) | -0.09 | 0.91 (0.67, 1.25) | .58 |  | ... | ... | ... |
| α-fetoprotein (ng/mL) | 0.00 | 1.00 (1.00, 1.00) | .34 |  | ... | ... | ... |
| α-fetoprotein (ng/mL)^†^ | 0.12 | 1.13 (1.07, 1.19) | **<.001** |  | ... | ... | ... |
| α-fetoprotein (>100 ng/mL vs ≤100 ng/mL) | 0.50 | 1.65 (1.26, 2.15) | **<.001** |  | ... | ... | ... |
| α-fetoprotein (>400 ng/mL vs ≤400 ng/mL) | 0.54 | 1.71 (1.28, 2.28) | **<.001** |  | ... | ... | ... |
| **Surgical** | | | |  |  |  |  |
| Open surgery (yes vs no) | 0.30 | 1.35 (1.00, 1.82) | .05 |  | ... | ... | ... |
| Major hepatectomy (yes vs no) | -0.02 | 0.98 (0.63, 1.51) | .91 |  | ... | ... | ... |
| **Histopathologic** |  |  |  |  |  |  |  |
| Microvascular invasion (present vs absent) | 0.83 | 2.30 (1.77, 3.00) | **<.001** |  | 0.78 | 2.18 (1.68, 2.84) | **<.001** |
| Edmondson-Steiner grade (III/IV vs I/II) | 0.13 | 1.14 (0.86, 1.51) | .37 |  | ... | ... | ... |
| Note.—Data in parentheses are 95% CIs. After accounting for multicollinearity, 12 variables (early washout, tumor size >5, mosaic architecture, blood products in mass, necrosis or severe ischemia, intratumoral artery, non-smooth tumor margin, tumor growth subtype, involvement of liver capsule, age, α-fetoprotein†, microvascular invasion) underwent multivariable analysis. sHR = subdistribution hazard ratio. †Data were computed after logarithmic transformation. | | | | | | | |

**Table S14. Frequencies of different nonperipheral washout patterns according to clinical and imaging factors that may impact image interpretations**

| **Factors** | **Conventional nonperipheral washout** | | | **Nonperipheral washout pattern** | | | | **Early Washout** | | |
| --- | --- | --- | --- | --- | --- | --- | --- | --- | --- | --- |
|  | Absent | Present | ***P* value** | No washout | Late washout | Early washout | ***P* value** | Absent | Present | ***P* value** |
| ***MRI field strength*** | | | | | | | | | | |
| 1.5 Tesla (n=310) | 62 (20.0) | 248 (80.0) | **.01** | 62 (20.0) | 58 (18.7) | 190 (61.3) | **.01** | 120 (38.7) | 190 (61.3) | .53 |
| 3.0 Tesla (n=301) | 87 (28.9) | 214 (71.1) |  | 87 (28.9) | 37 (12.3) | 177 (58.8) |  | 124 (41.2) | 177 (58.8) |  |
| ***Cirrhosis*** | | | | | | | | | | |
| Absent (n=239) | 57 (23.9) | 182 (76.2) | .80 | 57 (23.9) | 37 (15.5) | 145 (60.7) | .97 | 94 (39.3) | 145 (60.7) | .81 |
| Present (n=372) | 92 (24.7) | 280 (75.3) |  | 92 (24.7) | 58 (15.6) | 222 (59.7) |  | 150 (40.3) | 222 (59.7) |  |
| ***Liver steatosis*** | | | | | | | | | | |
| Absent (n=498) | 113 (22.7) | 385 (77.3) | **.04** | 113 (22.7) | 86 (17.3) | 299 (60.0) | **.02** | 199 (40.0) | 299 (60.0) | .98 |
| Present (n=113) | 36 (31.9) | 77 (68.1) |  | 36 (31.9) | 9 (8.0) | 68 (60.2) |  | 45 (39.8) | 68 (60.2) |  |
| ***Enhancing capsule*** | | | | | | | | | | |
| Absent (n=33) | 24 (72.7) | 9 (27.3) | **<.001** | 24 (72.7) | 4 (12.1) | 5 (15.2) | **<.001** | 28 (84.8) | 5 (15.2) | **<.001** |
| Present (n=578) | 125 (21.6) | 453 (78.4) |  | 125 (21.6) | 91 (15.7) | 362 (62.6) |  | 216 (37.4) | 362 (62.6) |  |
| ***Mosaic architecture*** | | | | | | | | | | |
| Absent (n=475) | 138 (29.1) | 337 (71.0) | **<.001** | 138 (29.1) | 87 (18.3) | 250 (52.6) | **<.001** | 225 (47.4) | 250 (52.6) | **<.001** |
| Present (n=136) | 11 (8.1) | 125 (91.9) |  | 11 (8.1) | 8 (5.9) | 117 (86.0) |  | 19 (14.0) | 117 (86.0) |  |

Note.— Data are presented as number (percentage).

*P* values were derived from the Chi-square test comparing the distribution across subgroup.
